# Personal data protection compliance assessment: A privacy policy scoring approach and empirical evidence from Thailand's SMEs

**DOI:** 10.1016/j.heliyon.2023.e20648

**Published:** 2023-10-17

**Authors:** Panchapawn Chatsuwan, Tanawat Phromma, Navaporn Surasvadi, Suttipong Thajchayapong

**Affiliations:** National Electronics and Computer Technology Center (NECTEC), NSTDA, 111 Phahonyothin Road, Khlong Nueng, Khlong Luang, Pathum Thani, 12120, Thailand

**Keywords:** Privacy policy, Scoring model, Personal data protection, PDPA, Small and medium-sized enterprises, SMEs

## Abstract

*Privacy policies*, intended to provide information to individuals regarding how their personal data is processed, are often complex and challenging for users to understand. Businesses often demonstrate non-compliance with personal data protection laws, ranging from the absence of privacy policies to the existence of policies that do not adhere to legal requirements. This paper aims to (1) develop a quantitative and systematic tool for evaluating privacy policies' compliance with the Personal Data Protection Act (PDPA), (2) assess compliance among Small and Medium Enterprises (SMEs) in Thailand, and (3) provide recommendations for enhancing compliance practices. To achieve this, we proposed a multi-criteria *privacy policy scoring model* integrated with comprehensive statistical data analyses. The privacy policy scoring model consists of ten privacy principles and 31 privacy criteria, providing a structured framework for evaluating privacy policies. During a two-year postponement period for enforcing the PDPA law, we conducted a stratified random-sampling survey of 384 SMEs to evaluate their privacy policies using the proposed scoring model. The accomplished results revealed significantly lower scores than anticipated, with the nationwide average score of SMEs reaching only 6.1909 out of 100 points. More than half of the SMEs collected personal data without announcing privacy policies, and those with privacy policies adhered to an average of only 12.15 out of 31 privacy criteria. These findings highlight the pressing need to improve compliance practices among SMEs in Thailand. The proposed methodology can be customized and applied to align with the requirements of personal data protection laws in other countries. Additionally, our findings indicate that compliance with the PDPA is influenced by the Thailand Standard Industrial Classification (TSIC) sections, suggesting the adoption of tailored approaches by policymakers to address the specific needs of different TSIC sections.

## Highlights

• Introducing a *privacy policy scoring model* to score privacy policies for compliance with a personal data protection law.
• The model offers a systematic and quantitative tool for businesses and policymakers to assess and decide on PDPA compliance.
• Findings from a survey of 384 Thai SMEs revealed a generally poor level of PDPA compliance in their privacy policies.
• Most SMEs did not provide opt-out options, consent forms, consent withdrawal mechanisms, or non-necessary cookie rejection.
• TSIC sections influenced PDPA compliance, recommending policymakers adopt tailored approaches for different sectors.

## 1 Introduction

Privacy has significantly become a subject of growing concern to the international community as the world population has increasingly accessed information and services online [1], [2], [3], [4]. As a result of technological advancement, the acquisition and usage of personal data have become more widespread and easily accessible. Websites and service providers often collect large amounts of personal data, which can be aggregated and utilized for creating and learning personal profiles. This raises concerns that the data may be used for unauthorized purposes, potentially causing harm to individuals [5]. While cases of personal data infringement (e.g., identity theft, tracking, stalking, and misuse of personal data) can induce negative credibility to businesses [6], customers themselves are less concerned about their privacy and willing to share their personal data if they are confident that infringement can be prevented [7]. In terms of loss, infringement of personal data protection has also been reported to cause enormous damage to the global economy by $6 trillion globally in 2021 [8]. In Thailand, Electronic Transactions Development Agency (ETDA) has revealed that personal data were at risk of identity theft, data processing, profiling, exploitation, and misuse for marketing, tracking, and stalking purposes [9].

At the international level, several regulatory frameworks, such as the Fair Information Practice Principles (FIPPs) and the recent EU's General Data Protection Regulation (GDPR), recommend and mandate companies to provide users with a *privacy notice* to inform them how companies collect, store, and manage their personal information [10]. The GDPR was enacted on May 25th, 2018, as the legal framework that sets guidelines for collecting and processing EU people's personal information [11].

Drafting based on GDPR's principles, the Personal Data Protection Act, B.E. 2562 (2019)(PDPA) in Thailand has been approved by the cabinet to come into force partially on May 28th, 2019. However, its full enforcement has been postponed for two years, until recently in effect on June 1st, 2022. The PDPA has designated individual persons as data subjects and all sectors, especially businesses, as data controllers or data processors. Consequently, awareness of data privacy protection roles as data subjects, data controllers, and data processors is of the utmost importance. According to Thailand's PDPA, online businesses are required to announce a *privacy policy* on their websites outlining how they collect, store, and manage customers' personal data.

Research on privacy policy assessment has been conducted in various regions worldwide, including China [12], EU [13], [5], [10], and South Asian [14], from both government [13], [5], [12] and business [14] contexts. Previous studies primarily involved surveying websites to evaluate compliance of privacy policies with personal data protection laws in each country based on predetermined privacy principles [5], [10], [15], [13], [12], [14], [16]. Methods employed in these studies included interviews [15] and surveys conducted by manual assessment [13], [12], [14], web content analysis [5], web scraping [10], machine learning [10], or application-based analysis [10], [16]. In these studies, the analysis of privacy policy content was given significant importance in order to determine adherence to required legal aspects. Privacy principles used for evaluation in these works were predetermined through a literature review or expert consultations. Overall, findings in these studies showed a lack of attention to privacy statements, especially in government agencies [13], [5], [12], inadequate information in privacy policies [5], [12], and a lack of clarity in privacy policies [15].

The objective of this paper is to develop a quantitative and systematic tool for evaluating privacy policies' compliance with Thailand's PDPA and to assess compliance among Small and Medium Enterprises (SMEs) in Thailand. While the previous studies mentioned had their own predetermined privacy principles for assessing privacy policies, these principles were defined at a high level or based on a set of keywords, lacking a clear and well-defined scoring assignment at a detailed level like the privacy criteria outlined in our work. The explicit consideration of privacy criteria within each privacy principle adds granularity and depth of analysis to our scoring model, allowing us to capture the finer details within each privacy principle. Furthermore, in the previous studies, the assessment of privacy policies was conducted by considering each privacy principle independently, without the ability to calculate a single numeric score based on weighted privacy criteria. This made it challenging to compare and evaluate privacy policies comprehensively.

Our approach addresses these limitations by developing a *privacy policy scoring model* based on a Multi-Criteria Decision Making (MCDM) method [17], [18] that encompasses both the high-level privacy principles and the detailed privacy criteria within each specific privacy principle. We consider the specific privacy criteria and their associated weights, allowing for a more nuanced and comprehensive evaluation of privacy policies. This allows a more accurate and meaningful comparison of businesses' privacy policies based on a single numeric score. By incorporating the well-defined privacy criteria and their relative weights, our scoring model provides decision-makers with a clear and structured framework for evaluating privacy policies, enabling informed and well-balanced decisions based on a comprehensive assessment of the high-level privacy principles and detailed privacy criteria. Additionally, the significance of SMEs has been disregarded in past research on privacy policy assessment. Our empirical study addresses this gap by providing insights into this neglected area of literature. The main contributions of the paper can be summarized as follows:

1. Introducing a novel *privacy policy scoring model* for evaluating PDPA-compliant privacy policies, which, to our knowledge, is the first of its kind. This scoring model offers a systematic and quantitative tool that benefits two key groups:
  (a) For business owners, the model provides a clear and well-defined checklist to assess the compliance of their privacy policies with PDPA, enabling them to identify areas for improvement.
  (b) For policymakers and government authorities, the scoring model offers a tool to evaluate businesses in Thailand, providing insights into the current state of PDPA compliance and facilitating informed decisions to promote personal data protection awareness among the appropriate audience.
2. Demonstrating the practical implementation of the scoring model by evaluating the privacy policies of a sample group of Thai SMEs. Comprehensive statistical data analyses were employed to gain insights into the PDPA compliance situation of Thai SMEs. This empirical study fills a gap in the literature as previous research on privacy policy assessment has overlooked SMEs, despite their significant economic role. This research sheds light on SMEs' privacy policy practices, contributing to a more comprehensive understanding of the implementation of personal data protection across various sectors.
3. Providing actionable steps derived from the proposed scoring model by offering recommendations to SMEs and government agencies on enhancing PDPA compliance based on the empirical findings.

While the focus of this paper is to assess compliance with Thailand's PDPA law, we anticipate that the methodology used to develop the scoring model, along with the extensive statistical analyses applied to evaluate the survey results, can be adapted and applied to meet the needs of personal data protection laws in other regions through a few key steps:

1. The scoring model can be customized by adjusting the definition and score assignment of the privacy criteria according to relevant data protection laws of the target region and updating the weights of the privacy criteria to reflect decision-makers' preferences.
2. A similar stratified random-sampling survey can be conducted among organizations in the target region to evaluate their privacy policies using the adapted scoring model derived in the previous step.
3. Descriptive statistical analyses and inferential statistical analyses, including the Kruskal-Wallis test [19], [20], [21], [22], [23] and the Dunn post hoc test [24], [21], [25], can be applied to analyze the survey results to assess compliance with local data protection laws and compare compliance among different sectors.

In conclusion, the methodology can be tailored and implemented by following these steps to ensure compliance with personal data protection laws in various regions.

The remainder of this paper is organized as follows. Firstly, Section 2 presents background and related work. Section 3 describes the methodology. Then, analysis results are presented in Section 4. Recommendations for SMEs and policy recommendations for government agencies are discussed in Section 5. Section 6 outlines limitations and future directions. Finally, Section 7 concludes the paper.

## 2 Background

This section provides an overview of the background relevant to the development of the *privacy policy scoring model* and discusses our motivation for choosing Thai SMEs as the research subjects.

### 2.1 GDPR

General Data Protection Regulation (GDPR)^1^ is a regulation introduced in 2016 for the protection of personal data of citizens in member countries of the European Union (EU). The scope of GDPR extends beyond European companies and includes global private sectors that engage in data exchange or communication with European companies and collect personal data of EU citizens. GDPR allows EU people to control their personal data and requires service providers to efficiently store customers' personal data. Individuals can request data controllers to delete, copy, or correct their personal data, and service providers must adhere to the requests.

### 2.2 Thailand's personal data protection act (PDPA)

The PDPA is a general law in Thailand for protecting personal data and imposes obligations on both public and private sectors to ensure compliance. It is considered one of the strongest data privacy laws in Asia and surpasses Thailand's previous legislation, the 2001 Electronic Transactions Act [26]. The PDPA incorporates several data protection principles from the GDPR and is on par with other international standards.

*Personal data* under the PDPA refers to information that can directly or indirectly identify an individual. Examples of personal data include name, surname, identification number, address, telephone number, date of birth, gender, education, occupation, photograph, and financial information. Additionally, personal data may include sensitive information such as blood group, religion, and congenital disease. However, personal data excludes information about deceased individuals [27].

#### 2.2.1 Overview of PDPA

This section summarizes the essence of Thailand's PDPA, covering its key aspects such as the purposes of the PDPA, lawful bases for collecting, using, or disclosing personal data, and relevant parties.

##### Purposes of PDPA

1. To protect personal data by establishing standards and mechanisms that appropriately govern its collection, usage, disclosure, and prevention of misrepresentation or unauthorized access.
2. To prevent and solve problems in violation of personal data rights and to ensure that data subjects are protected and able to monitor and control the collection, usage, and disclosure of their personal data by data processors. Besides, data subjects can accuse data processors in cases of infringement of the stated data processing purposes.

##### Lawful bases for collecting, usage, or disclosure of personal data

The collection, usage, or disclosure of personal data must be done under at least one of the following principles: (1) legal obligations, (2) legitimate interest, (3) public task, (4) contract, (5) vital interest, (6) scientific or historical research, and (7) consent.

##### Relevant parties

1. *Data subject* is the person to whom the information is addressed. The owner of the personal data has the following rights: the right to be informed, the right to request access to personal data, the right to receive and transfer, the right to object, the right to request deletion, the right to withdraw consent, the right to request data suspension, and the right to request correction of information.
2. *Data controller* is a person or juristic person with the authority to make decisions about collecting, using, or disclosing personal data in compliance with the PDPA.
3. *Data processor* is a person or juristic person who controls the system or performs the commands of the data controller. The data processor may or may not be the same as the data controller.
4. *The Personal Data Protection Committee* is responsible for ensuring that all sectors in Thailand comply with PDPA. In the event of a data breach or any infringement, violators will face civil, criminal, and administrative penalties.

### 2.3 Privacy policy

A *privacy policy*, also referred to as a *privacy notice* or *privacy statement*, is a statement established to inform data subjects about the details and purposes of data processing. It serves as the primary medium of information dissemination between the data controller and the users. Privacy policies are commonly announced on websites and should be easily accessible to anyone. GDPR and PDPA mandate that data controllers provide data subjects with an independent page of their privacy policy and inform data subjects before or during the collection of personal data. Organizations benefit from the privacy policy as it helps to build their credibility and trust in data protection and security and ensure the appropriate usage of personal data for its intended purposes.

The visibility of the privacy notice not only affects the user's risk perception [28] but also plays a role in building trust in the website [29], [30]. However, many websites provide low visibility of privacy policies and offer limited choices for personal data collection. Most businesses have yet to provide clear and concise privacy policies [15]. Consequently, users remain concerned about privacy due to the legal context and technical jargon used in privacy policies, even though businesses comply with personal data protection regulations as announced in their privacy policies [31]. Businesses can improve customer trust and reduce negative attitudes towards the business by giving customers a sense of ownership over their own data [7], [32]. Therefore, it is crucial for businesses to have a clear, concise, and noticeable privacy policy to gain customer trust and establish a positive attitude toward the business's processing and storage of personal data.

### 2.4 Cookie policy

*Cookies* are small text files containing data that websites send to the browsers of their visitors. Cookies assist websites in remembering information about visitors, making visitors revisit the site more easily or making the website more beneficial to them. Internet advertisements often use cookies to help businesses better understand their target customers' behaviors or to manage online behavioral advertising. A *Cookie Policy* is a document that informs data subjects about how data processors use cookies, what types of cookies will be collected for the purposes of personal data processing, and how long cookies will remain stored on visitors' computers.

While most customers are concerned about the use and disclosure of cookies on websites that track their browsing behaviors, it is the responsibility of websites to allow consumers to disagree on tracking. However, some customers willingly consent to tracking in exchange for privileges, price updates, or promotions. In practice, however, many websites track customers without obtaining their consent [33], and some collect cookies before any consent is given. Additionally, in order to collect consumers' insights, there are various strategies or “dark patterns” used in cookie consent [34], [35] to present deceiving information and continue tracking customer behavior [36]. After the enforcement of GDPR, web tracking declined or stopped after customers opted out; nonetheless, some websites keep tracking once the website is loaded [37]. This suggests that there is a gap between regulations and practices, with many websites disregarding privacy regulations and installing cookies before consent is obtained [38]. Implementing a cookie policy, disclosure statement, cookie consent, or similar notice can help reduce users' concerns and negative reactions, benefiting all parties involved [39].

### 2.5 SMEs

Small and Medium-sized Enterprises (SMEs) are the economic backbone of almost every economy in the world. SMEs represent more than 95% of registered firms worldwide, generate more than 50% of employment opportunities, and contribute over 35% to the GDP in many emerging markets [40]. In the APEC region, SMEs contribute significantly to economic growth, with their main share of GDP ranging from 40% to 60% [41]. Thailand, the second-largest economy in Southeast Asia, has consistently achieved robust economic growth thanks to the country's solid foundations and diverse industries of SMEs [42].

SMEs are a driving force in Thailand's economy, representing the majority of firms in the country [43]. In 2020, SMEs constituted 99.54% of all enterprises in Thailand and contributed 34.2% to Thailand's GDP in 2021 [44]. SMEs also play a crucial role in linking different industrial units and filling gaps in industrial clusters that may not be covered by larger enterprises [35]. Presently, 98.4% of Thai entrepreneurs use the Internet for business, and 67.4% of Thai individuals primarily engage in online transactions, such as buying and selling products and services, through the Internet. Approximately 38.6% of SMEs have an online distribution channel, and 79.3% of SMEs offer online payment services [44]. Therefore, it becomes crucial for both businesses and customers to be aware of the protection of personal information online [45].

In addition to the significance of SMEs mentioned above, Thai SMEs have faced various challenges, including limited digital literacy, lack of knowledge about business laws, and budget constraints for implementing personal data protection measures. Some SMEs also struggle with understanding the guidelines for PDPA compliance, adding to the complexity. This paper thus selected Thai SMEs as research subjects to demonstrate an application of the developed *privacy policy scoring model* and acquire insights into the PDPA compliance state among Thai SMEs. The outcomes of the SME assessment could aid policymakers in formulating policies to foster PDPA awareness and compliance and supporting SMEs in understanding the basics of PDPA compliance.

Furthermore, to the best of our knowledge, previous studies on privacy policy assessment have not specifically targeted SMEs, despite their vital role in the economy. Therefore, this research fills a gap in the literature by examining the privacy policy practices of SMEs, shedding light on their compliance status, and contributing to a more comprehensive understanding of the implementation of personal data protection across various sectors.

#### 2.5.1 SMEs definition in Thailand

In Thailand, SMEs are defined under the SMEs Promotion Act (No.2) based on the number of employees and income criteria specified in the Ministerial Regulations, as presented in Table 1 [46].

**Table 1:** The categorization of small and medium-sized enterprises.

Sectors	Small and Micro				Medium	
	Micro		Small			
	**Table:** The number of employees	**Table:** Income (Million Baht)	**Table:** The number of employees	**Table:** Income (Million Baht)	**Table:** The number of employees	**Table:** Income (Million Baht)
Manufacturing	≤5	≤1.8	≤50	≤100	51 − 200	100 − 500
Trade and Services	≤5	≤1.8	≤30	≤50	31 − 100	50 − 300

#### 2.5.2 SMEs categorized by enterprise sectors

SMEs can be categorized into four groups based on their enterprise sectors as follows:

1. *Manufacturing sector*: This group includes businesses involved in the production of industrial and mining products, as well as those engaged in the transformation of raw materials into finished products.
2. *Trade sector*: SMEs in this category focus on import and export activities, as well as wholesale and retail operations. However, it does not encompass manufacturing businesses.
3. *Service sector*: This sector includes SMEs that provide various services such as education, healthcare, transportation, food or beverage sales, hotel services, insurance, and beauty salon.
4. *Agricultural sector*: Businesses involved in the utilization of natural resources, including cultivation, animal production, hunting, and related services, fall into this category.

#### 2.5.3 SMEs categorized by Thailand industry standard classification 2009 (TSIC)

The Thailand Industry Standard Classification 2009 (TSIC) system is used to group and assign codes to economic activities in Thailand. It is based on the International Industry Standard Classification (ISIC) Rev.4 system of the United Nations, which organizes activities by economic structure. The ISIC Rev.4 can be applied to various economic structures, statistical activities, and the specific needs of different countries. It serves multiple purposes, including labor management, career guidance, industry statistics collection, and cross-country data comparison [47]. Similar to ISIC Rev.4, TSIC classifies similar industries into the same category and hierarchically divides categories into four levels: *Sections*, *Divisions*, *Groups*, and *Classes*. The *TSIC Section* is the highest level, consisting of 21 sections represented by capital letters from A to U. The Department of Employment, Ministry of Labor in Thailand has classified SMEs by TSIC Section [47], as shown in Table 2.

**Table 2:** Thailand Industry Standard Classification 2009 (TSIC) [47].

TSIC Section	Description
A	Agriculture, forestry, and fishing
B	Mining and quarrying
C	Manufacturing
D	Electricity, gas, steam, and air conditioning supply
E	Water supply, sewerage, waste management, and remediation activities
F	Construction
G	Wholesale and retail trade, repair of motor vehicles and motorcycles
H	Transportation and storage
I	Accommodation and food service activities
J	Information and communication
K	Financial and insurance activities
L	Real estate activities
M	Professional, scientific, and technical activities
N	Administrative and support service activities
O	Public administration and defense, compulsory social security
P	Education
Q	Human health and social work activities
R	Arts, entertainment, and recreation
S	Other service activities
T	Household employment activities; self-produced production and service activities for household use, which cannot be clearly categorized
U	Activities of international organizations and associations.

## 3 Methodology

**Figure 1:**
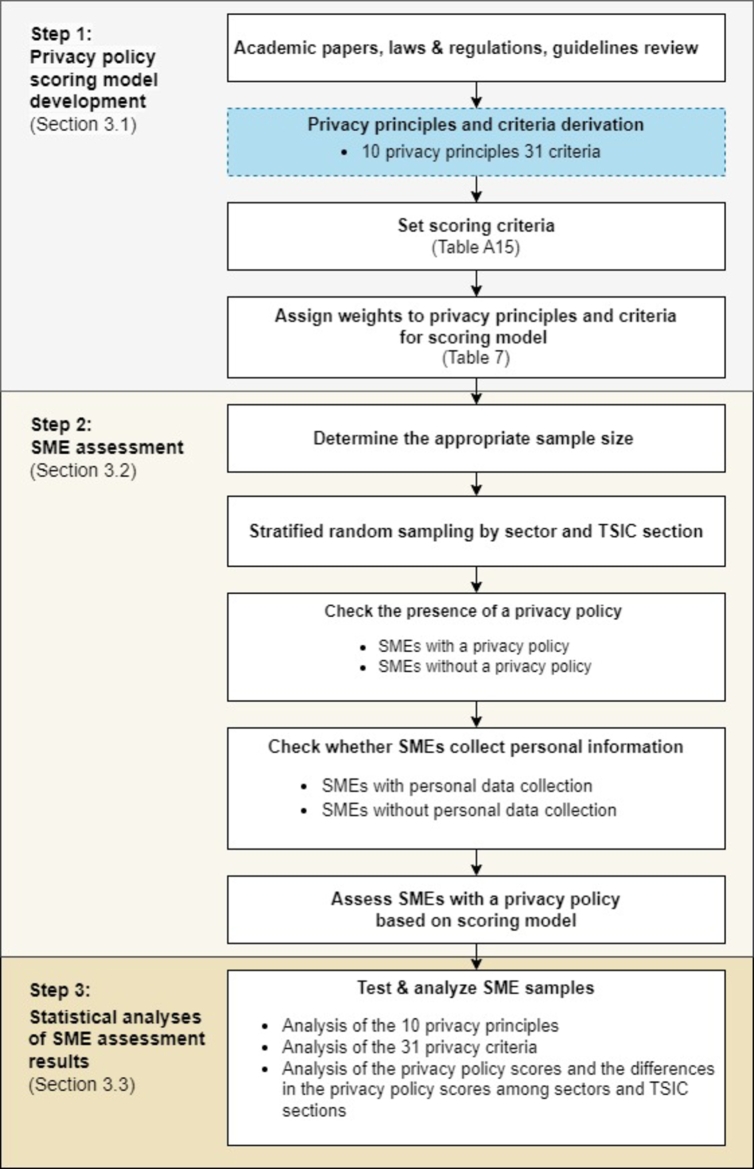
The overall process of the paper. The *privacy principles and criteria derivation* step (shown in the dotted box) is expanded to describe in more detail in Fig. 2.

This study aims to develop a *privacy policy scoring model* for evaluating the compliance of businesses' privacy policies with the PDPA in Thailand and to apply it to assess Thai SMEs. The overall process of the paper is outlined in Fig. 1.

There are three main analysis steps in this work as follows.

***Step 1:** Privacy policy scoring model development.* The *privacy policy scoring model* proposed in this work aims to provide a systematic and quantitative approach for evaluating and comparing businesses' privacy policies with Thailand's PDPA. The proposed scoring model encompasses ten *privacy principles* and 31 *privacy criteria*. These ten *privacy principles* cover essential elements of privacy policies that align with international laws and academic research. The 31 *privacy criteria* are specifically tailored to align with Thailand's PDPA law requirements, addressing aspects such as data collection, processing, storage, sharing, user consent, child privacy, and security measures. These privacy principles and criteria are derived from a content analysis [48]. Each criterion is assigned a weight to reflect its relative importance in the overall evaluation process. To calculate the privacy policy scores, the model assigns scores to different sections or clauses of the privacy policy based on their alignment with the specific criterion. The scores are then aggregated using the weighted sum method, resulting in an overall score representing the privacy policy's quality and comprehensiveness.

***Step 2:** SME assessment.* To demonstrate the application of the scoring model obtained in Step 1 and examine the PDPA compliance situation among Thai SMEs, a stratified random sampling method [49] was employed. The sample size of 384 SMEs was determined using Cochran's formula [49] at a 95% confidence level and ±5% precision. These SMEs were selected from the larger population of Thai SMEs and subsequently evaluated based on the scoring model.

By selecting a sample size determined by Cochran's formula, the study aimed to achieve a statistically significant sample that would provide reliable and accurate insights into the overall compliance situation. The stratified random sampling approach ensured representative coverage across different strata of SMEs, including sectors and TSIC sections.

The selected SMEs were assessed using the scoring model derived in Step 1. The evaluation involved analyzing the SMEs' privacy policies, where each criterion was assigned a score according to the extent of compliance exhibited by the SME.

***Step 3:** Statistical analysis of SME assessment results.* In this last step of the study, several statistical analyses are conducted on the SME assessment results obtained in Step 2. These analyses involve descriptive and inferential statistics, aiming to gain insights into the PDPA-compliance situation among Thai SMEs and provide guidance for policy recommendations and support mechanisms to improve compliance efforts. The findings from these statistical analyses contribute to understanding the current state of PDPA compliance among Thai SMEs. They provide insights into the areas where SMEs may fall short in compliance, highlight trends or patterns in compliance levels, and inform policy recommendations and support mechanisms to enhance compliance efforts.

Each of these steps is described in more detail in subsequent sections.

### 3.1 Privacy policy scoring model development

This section describes our approach to developing a *privacy policy scoring model*, consisting of 10 *privacy principles* and 31 *privacy criteria*, through a content analysis [48] of laws, regulations, academic papers, and related documents. Detailed steps involved in the development process are shown in the upper part of Fig. 1 and are as follows.

1. Firstly, to obtain the main *privacy principles* for the scoring model that can represent the universal view of privacy principles from both legal and practical perspectives, we examined the GDPR and academic papers. We gathered all available privacy principles and then categorized similar concepts into the same principle, resulting in a total of ten main privacy principles. Details of the derivation of these ten privacy principles are shown in the left pathway of Fig. 2.
  (a) From the viewpoint of international privacy policy laws and standards, we studied the GDPR and deduced seven main privacy principles, as shown in Table 3.

**Table 3:** Principles of GDPR.

Principles of GDPR^2^	Description
1. Lawfulness, fairness, and transparency	It is essential to clearly identify the valid grounds for processing personal information in order to ensure compliance with the law. The Common Information Security Control identifies six justifications for processing personal data, at least one of which must be present in order to meet the necessary requirements: consent, contract, legal obligation, vital interests, public task, and legitimate interests.

2. Purpose limitations	By following this concept, companies can ensure that data subjects know the reasons for gathering their own data and have sensible assumptions regarding how the company expects to manage it. Further, it provides data subjects some control over how their personal information is used in the future and allows them to decide whether they are willing to provide it.

3. Data minimization	The GDPR requires companies only to collect and retain the minimum amount of data necessary to fulfill their specific purpose. The regulation stresses the importance of collecting data that is relevant, necessary, and required. It also prohibits the practice of collecting data without a clear purpose, only in case it may be useful in the future. However, it does allow for the collection of data in anticipation of a known future need.

4. Accuracy	The GDPR requires companies only to collect and retain the minimum amount of data necessary to fulfill their specific purpose. The regulation stresses the importance of collecting data that is relevant, necessary, and required. It also prohibits the practice of collecting data without a clear purpose, only in case it may be useful in the future. However, it does allow for the collection of data in anticipation of a known future need.

5. Storage limitation	The GDPR stipulates that personal data should only be kept for as long as is necessary for the purpose for which it was collected. The regulation does not provide specific timeframes for data retention, and it is up to the companies to justify the length of time that they retain data. The longer the retention period, the greater the likelihood that the data will become inaccurate or outdated.

6. Integrity and confidentiality	This need extends beyond Internet security and also incorporates official and physical security. According to GDPR, only those with the appropriate authorization are allowed to access and manage personal data. Besides, if personal data is unexpectedly lost, changed, or crushed at any point, there is a way to recover it, removing the potential for any issues for data subjects.

7. Accountability	This part of GDPR requires those handling individual data to get a sense of ownership through their interactions with that data and their adherence to various criteria. The two measures and records should be set up to show consistency to accomplish this prerequisite. Additionally, it means that in the event of a problem, such as a data breach, it will usually be demonstrated that safeguards and measures were put in place to minimize the likelihood of such an occurrence. This could imply that there is a release from any legal authorization activity.
  (b) From a pragmatic standpoint, we reviewed academic papers that investigated *privacy policy*, *privacy notice*, and *privacy statement* published on websites from diverse countries, including EU countries, China, and South Asian countries. We acquired a total of 17 privacy principles, as shown in Table 4.

**Table 4:**
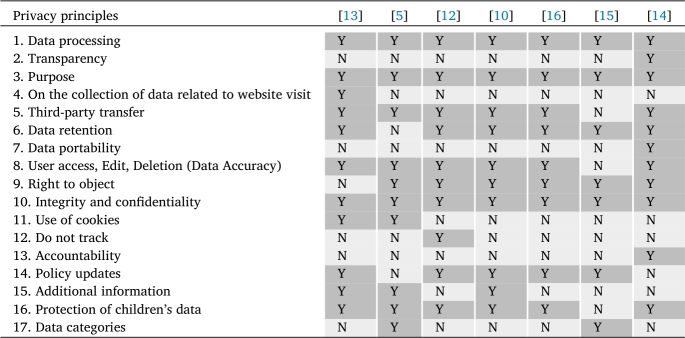
Privacy principles from a review of academic papers.
  (c) Given the seven GDPR privacy principles and 17 privacy principles from academic papers, we aligned the principles from papers (shown in the second column of Table 5) with those of the GDPR (shown in the first column of Table 5) based on similar definitions, if possible. Although some principles from academic papers did not entirely fit into the definitions of GDPR principles, we decided to keep them as they could potentially offer practical and helpful guidelines for implementation. As a result of this step, we obtained ten groups of privacy principles.

**Table 5:** Ten privacy principles collectively compiled from GDPR and literature review.

**GDPR**	Literature review	10 privacy principles
**Table:** 1. Lawfulness, fairness, and transparency 2. Purpose limitations 3. Data minimizations	**Table:** 1. Data processing 2. Transparency 3. Purpose 4. On the collection of data related to website visit	**Table:** 1. Data processing (Collection, Purpose) / Information use / On data processing and usage / Processing entities / First party collection or usage

N/A	5. Third-party transfer	**Table:** 2. Information disclosure / Third-party transfer/ On the disclosure of personal data to third parties / Third party sharing or collection

4. Storage limitation	**Table:** 6. Data retention	**Table:** 3. Data retention / Storage and retention of collected data

5. Accuracy	**Table:** 7. Data portability 8.User access, Edit & Deletion (Data Accuracy) 9. Right to object	**Table:** 4. Rights of data subjects: User Access, Edit, Delete, Object, Data accuracy and control, Data portability

6. Integrity and confidentiality	**Table:** 10. Integrity and confidentiality	**Table:** 5. Data protection / Data security / Security measures / Security of personal data / Integrity and confidentiality

N/A	**Table:** 11. Use of cookies 12. Do not track	**Table:** 6. User controls / User choice / Control / Opt-out option

		**Table:** 7. Policy change / Revisions in the privacy statements / Policy updates
7. Accountability	**Table:** 13. Accountability 14. Policy updates 15. Additional information	**Table:** 8. Privacy contact information / Additional information / Contact possibilities for inquiries regarding the privacy policies

N/A	16. Protection of children's data	9. International and specific audiences

N/A	17. Data categories	10. Data categories / Collected information
  (d) The ten groups acquired in the previous step were assigned privacy principle names according to keywords frequently appearing in the GDPR and academic papers. The final ten privacy principles are shown in the last column of Table 5.

**Figure 2:**
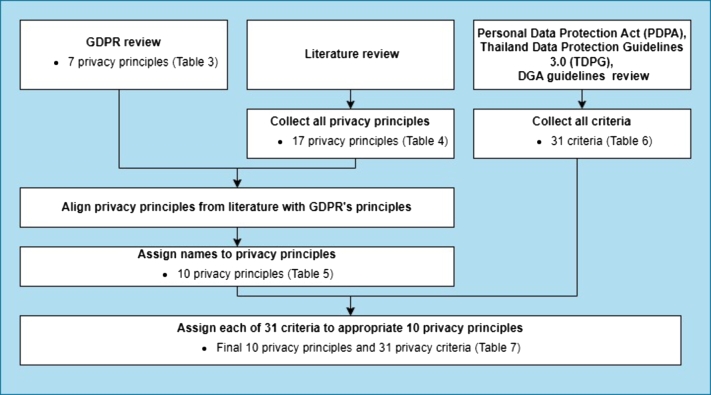
Privacy principles and criteria derivation.
2. Secondly, since the developed scoring model aims to evaluate Thai businesses' privacy policies, we inspected Thailand's PDPA-related documents, including (1) Thailand's PDPA law [27], (2) Thailand Data Protection Guidelines 3.0 (TDPG 3.0) [50], and (3) the privacy policy and cookie policy preparation guidelines by Digital Government Development Agency (DGA) [51]. This step enabled us to obtain 31 *privacy criteria* for the scoring model, as shown in Table 6. The derivation process of the 31 privacy criteria is shown in the right pathway of Fig. 2.

**Table 6:** 31 privacy criteria assembled from PDPA-related documents.

**List of privacy criteria**
1. Give meaning of personal data
2. Give meaning of cookies
3. Purpose of personal data collection
4. Data processing
5. Purpose of cookie collecting
6. Purpose of marketing promotion
7. Provide the first/third party cookie information
8. Sharing personal data with third party
9. Type of personal data collection
10. Storage and data retention period
11. Where the data will be stored
12. Cookie retention period
13. Accessibility
14. Data subject's rights
15. Cookie banner / Use of cookie / Notification of the usage of cookie
16. Provide link to cookie policy
17. Hypertext Transfer Protocol Secure (HTTPS)
18. How to protect the collected personal data
**Table:** 19. Statement notifying users of the limitation or scope of its liability when visiting other websites
20. Consent form
21. A channel or button for users to withdraw consent easily
22. Non-necessary cookie rejection
23. Cookie settings
24. Non-necessary cookie consent
25. How to disable cookies
26. If the policy changes, how data controller will do
27. Latest revision date
28. Contact information
29. Protection of children's data / Child privacy
30. Type of cookie collection
31. Where personal data come from / How to collect: directly or get from third party
3. Each of the 31 *privacy criteria*, derived from a national view in the previous step, was then assigned to one of the ten *privacy principles* derived from a universal view in the first step, based on the most similar definitions. Table 7 displays the final ten *privacy principles* and 31 *privacy criteria* for use in the scoring model.

**Table 7:** The final ten privacy principles and 31 privacy criteria for use in the scoring model and their associated weights.

Privacy principles	Privacy criteria	Weight
**Table:** 1. Data processing (Collection, Purpose)/ Information use / On data processing and usage / Processing entities / First party collection or usage	1. Give meaning of personal data	1/50
	2. Give meaning of cookies	1/50
	3. Purpose of personal data collection	1/50
	4. Data processing	1/50
	5. Purpose of cookie collecting	1/50

**Table:** 2. Information disclosure / Third-party transfer / On the disclosure of personal data to third parties / Third party sharing or collection	6. Purpose of marketing promotion	1/30
	**Table:** 7. Provide the first/third party cookie information	1/30
	8. Sharing personal data with third party	1/30

**Table:** 3. Data retention / Storage and retention of collected data	9. Type of personal data collection	1/40
	10. Storage and data retention period	1/40
	11. Where the data will be stored	1/40
	12. Cookie retention period	1/40

**Table:** 4. Rights of data subjects: User access, Edit, Delete, Object, Data accuracy and control, Data portability	13. Accessibility	1/40
	14. Data subject's rights	1/40
	**Table:** 15. Cookie banner / User of cookie / Notification of the usage of cookie	1/40
	16. Provide link to cookie policy	1/40

**Table:** 5. Data protection / Data security / Security measures / Security of personal data / Integrity and confidentiality	17. Hypertext Transfer Protocol Secure (HTTPS)	1/30
	18. How to protect the collected personal data	1/30
	**Table:** 19. Statement notifying users of the limitation or scope of its liability when visiting other websites	1/30

**Table:** 6. User controls / User choice / Control / Opt-out options	20. Consent form	1/60
	**Table:** 21. A channel or button for user to withdraw consent easily	1/60
	22. Non-necessary cookie rejection	1/60
	23. Cookie settings	1/60
	24. Non-necessary cookie consent	1/60
	25. How to disable cookies	1/60

**Table:** 7. Policy change / Revisions in the privacy statements / Policy updates	26. If the policy changes, how data controller will do	1/20
	27. Latest revision date	1/20

**Table:** 8. Privacy contact information / Additional information / Contact possibilities for inquiries regarding the privacy policies	28. Contact information	1/10

9. International and specific audiences	29. Protection of children's data / Child privacy	1/10

10. Data categories / Collected information	30. Type of cookie collection	1/20
	**Table:** 31. Where personal data come from / How to collect: directly or get from third party	1/20
4. To determine a privacy policy score of a business regarding PDPA compliance, a score of zero (0) or one (1) score was assigned to each of the 31 privacy criteria after inspecting the business's privacy policy. A score of zero indicated that the policy did not satisfy the required criteria, while a score of one indicated that the policy satisfied the criteria. Table A.15 in Appendix A describes the detailed scoring criteria.
5. A composite privacy policy score of a business was then calculated by the weighted sum of each criterion score according to Equation (1) discussed in Section 3.1.5.

#### 3.1.1 GDPR principles review

GDPR aims to provide data subjects with extensive control over their personal data collected by the controller companies. To be GDPR-compliant, companies must take careful measures to protect user data, such as pseudonymization and encryption. They should provide data subjects with ways to retrieve, delete, and object to the use of their data [15]. In Article 5 of the GDPR, there are seven principles: (1) Lawfulness, fairness, and transparency, (2) Purpose limitations, (3) Data minimization, (4) Accuracy, (5) Storage limitation, (6) Integrity and confidentiality, and (7) Accountability, where a description of each GDPR principle is shown in Table 3.

#### 3.1.2 Review of academic papers

A *privacy policy* is a primary communication channel between a data controller and data subjects to inform the details and purposes of data processing. Numerous studies have examined privacy statements on websites to analyze their content and compliance with relevant laws and regulations. Examples of such studies include investigations into privacy policies on municipal websites in the Netherlands [13], Chinese city government websites [12], Portuguese municipal websites [5], and South Asian websites [14]. These studies have found that privacy statements' availability, findability, and conformity varied greatly.

Beldad et al. [13] discovered that not all Dutch municipal websites had privacy policies, and most municipalities did not ensure that their online privacy statements were findable. They also found variations in the quality of privacy policies, with some containing all the essential provisions of the Dutch Personal Data Protection Act, while others were more general and vague. Dias et al. [5] studied the status of privacy policies advertised by Portuguese municipal websites and their compliance with advertised policies, aiming to increase awareness of such policies in local authorities in Portugal. Reddick and Zheng [12] examined the privacy protection of 100 cities in China through a benchmarking index and found that the overall performance of city governments in privacy statements was poor. Javed et al. [14] assessed website privacy policies in the three largest South Asian economies, finding low levels of accessibility, readability, and compliance with privacy principles, particularly in the education, healthcare, and government sectors.

The implementation of the GDPR in 2018 has also been shown to significantly impact privacy policies online, leading to a significant overhaul of policies both within and outside the EU [10]. In addition, Mohan et al. [15] found that many large-scale cloud services claiming GDPR compliance had unclear and potentially non-compliant privacy policies. The emergence of new forms of user interaction, such as voice, and the enforcement of new regulations like the GDPR pose challenges in providing privacy stakeholders with awareness and control [16]. Machine learning techniques have also been utilized in research to analyze privacy policies [16].

As shown in Table 4, this paper has compiled and grouped similar concepts of privacy principles used in examining privacy policies in the studies mentioned above. The table indicates whether each privacy principle was discussed in each paper using *Y* and *N*. Based on the seven GDPR privacy principles shown in Table 3 and 17 privacy principles from academic papers shown in Table 4, we aligned the privacy principles from academic papers with those of the GDPR based on similar definitions, whenever possible. However, certain principles from the academic papers could not be fully mapped to the GDPR principles but were retained as they offer valuable practical guidelines for businesses to follow. These principles include *Third-party transfer*, *Use of cookies*, *Do not track*, *Protection of children's data*, and *Data categories*. As a result of this step, we were able to organize the principles into ten distinct groups. The privacy principle names were assigned based on common keywords frequently appearing in GDPR and academic papers. These final ten privacy principles are presented in the last column of Table 5.

#### 3.1.3 Review of PDPA-related documents

The objective of this section is to identify a set of *privacy criteria* to be used in a scoring model for evaluating the privacy policies of Thai businesses. To achieve this, we have examined relevant documents related to Thailand's PDPA. Although the PDPA has been partially in effect since 2019, there is still a lack of widespread acknowledgment and adherence to the law. Additionally, there are complex issues related to the legal context of Thai policies that add complexity to the compliance landscape. Therefore, in this study, we have broadened our investigation beyond the PDPA and have also examined other relevant documents as follows:

1. *Thailand Data Protection Guidelines 3.0 (TDPG3.0)*[50] by the Faculty of Law, Chulalongkorn University provides essential guidelines for the implementation of personal data protection in accordance with the PDPA.
2. *Privacy policy and cookie policy preparation guidelines*[51] by the Digital Government Development Agency (DGA) offer organizations and businesses sample templates for drafting their own privacy and cookie policies that comply with the PDPA. The DGA template is a general privacy policy template that organizations in any domain can adopt.

The Faculty of Law, Chulalongkorn University, and DGA are reputable government agencies known for their legal and digital technology expertise, respectively. The Faculty of Law, Chulalongkorn University, offers legal services and advice to the public, while DGA provides standards, guidelines, measures, criteria, and methods for digital technology. To ensure the reliability and relevance of the identified privacy criteria, we conducted a comprehensive review with the input of legal and technology policy experts, carefully studying the guidelines provided by these two agencies. The results of this review are presented in Table 6.

A total of 31 *privacy criteria* were identified from a review of relevant documents. Each privacy criterion was then assigned to one of the ten *privacy principles* acquired from the review of GDPR and literature in Sections 3.1.1 and 3.1.2, based on their closet definitions. This process resulted in the final ten *privacy principles* and 31 *privacy criteria* for use in the *privacy policy scoring model*. These privacy principles and criteria are shown in the first and second columns of Table 7, respectively.

#### 3.1.4 Scoring criteria

In order to determine a privacy policy score of a business for PDPA compliance, an appraisal of the business's privacy policy is required to specify whether the policy satisfies each privacy criterion. Table A.15 in Appendix A presents a detailed description of each privacy criterion. A score of zero (0) was given to a criterion for which the business's privacy policy did not meet the specified description, while a score of one (1) was assigned to a criterion for which the policy fulfilled a description. That is, a score of zero indicates a non-PDPA-compliant criterion; on the other hand, a score of one represents a PDPA-compliant criterion.

#### 3.1.5 A privacy policy score calculation

Each business is assigned a privacy policy score according to its compliance with each of the 31 privacy criteria. The maximum privacy policy score is 100 percent, with each of the ten main privacy principles equally weighted, resulting in a maximum score of 10 percent or 1/10 for each privacy principle. Within each privacy principle, all privacy criteria are also weighted equally. For example, the third privacy principle—Data retention / Storage and retention of collected data—has four privacy criteria (criteria number 9 to 12). Therefore, each privacy criterion is assigned a weight of 1/40 or 2.5 percent.^3^ The last column of Table 7 shows the associated weights of 31 privacy criteria. A composite privacy policy score was then calculated by the weighted sum of each privacy criterion score according to the following equation:

(1) $$\text{Privacy policy score}=\sum_{i=1}^{31}X_{i}W_{i}\times 100$$

$$X_{i}=\text{a score of a criterion}\;i=\left\{ \begin{array}{cc} 1, & \text{if}\;\text{i}\;\text{complies with PDPA}\\ 0, & \text{if}\;\text{i}\;\text{does not comply with PDPA} \end{array}\right.$$

$W_{i}=$ a weight of a criterion *i* as shown in the last column of Table 7.

### 3.2 SME assessment

This section illustrates the application of the *privacy policy scoring model* developed in Section 3.1 to evaluate Thai SMEs. The detailed steps are presented in the middle part of Fig. 1.

#### 3.2.1 Coverage

The population for this research consisted of all SMEs in Thailand. According to the most recent data from the Office of SMEs Promotion (OSMEP), there were, in total, 3,134,442 SMEs in Thailand in 2020, classified by sectors and TSIC sections. The sectors include manufacturing, trade, service, and agricultural sectors. The TSIC consists of 21 TSIC sections according to Table 2 [44], [57]. The populations of SMEs in each sector and TSIC section are shown in the second column of Table 8 and Table 9, respectively.

**Table 8:** Proportional allocation of sample size for sector.

Sector	Population	Proportion (%)	Sample size
Manufacturing	532,104	16.98	65
Trade	1,288,256	41.10	158
Service	1,256,755	40.10	154
Agricultural	57,327	1.83	7

Total	3,134,442	100	384

Source: Sample size calculated by the authors based on population and proportion from [44].

**Table 9:** Proportional allocation of sample size for TSIC section.

TSIC section	Population	Proportion (%)	Sample size
A	57,327	1.83	7
C	518,843	16.55	64
F	132,559	4.23	16
G	1,288,256	41.10	158
H	74,916	2.39	9
I	367,618	11.73	45
L	181,369	5.79	22
M	76,444	2.44	9
N	77,911	2.49	10
S	250,016	7.98	31
*Other*	109,183	3.48	13

Total	3,134,442	100	384

Source: Sample size calculated by the authors based on population and proportion from [44].

#### 3.2.2 Sample size and design

To estimate a percentage or a proportion of Thai SMEs in various aspects regarding PDPA compliance, we determined an appropriate sample size for the survey according to Cochran's formula for a large population [49], given by

(2) $$n_{0}=\frac{z^{2}pq}{e^{2}}=\frac{{\left(1.96\right)}^{2}(0.5)(1-0.5)}{{\left(0.05\right)}^{2}}=384,$$

where $n_{0}$ is the appropriate sample size, *z* is the z-score of the standard Normal distribution at a given confidence level, *e* is the desired level of precision, *p* is the estimated proportion of an attribute of interest present in the population, and *q* is equal to 1−p. In the case that the value of *p* is unknown, we can assume p=0.5, which will yield the maximum sample size with the desired precision. In this work, a sample size of 384 was determined, as we desired a 95% confidence level and a precision of ±5%.

Furthermore, to provide better coverage of the SME population and to ensure each SME subpopulation has proper representation within the sample, we employed a stratified random sampling [49] approach. Initially, we divided the SME population into subpopulations according to sectors and TSIC sections, as mentioned earlier, and then applied random sampling methods to each SME subpopulation. For each SME subpopulation, we determined a sampling proportion computed by dividing the size of that subpopulation by the total population (i.e., proportionate allocation strategy). The proportions of SME subpopulations according to sectors and TSIC sections are presented in the third column of Table 8 and Table 9, respectively. The sample size for each SME subpopulation according to sectors and TSIC sections is shown in the last column of Table 8 and Table 9, respectively. To facilitate the statistical analysis of differences among groups, we consolidated the TSIC sections that did not rank among the top 10 (with a proportion less than 1.14%). These sections, specifically B, D, E, J, K, O, P, Q, R, T, and U, were combined and labeled as the *Other* section.

#### 3.2.3 Survey process

In December 2021, a survey was conducted to assess the privacy policies of Thai SMEs with respect to PDPA compliance. The survey procedures are outlined below.

1. We sampled SMEs from each sector and TSIC section according to the sample sizes determined in Table 8 and Table 9, respectively, using data from the Thai SME-GP platform (https://thaismegp.com/) and the DBD DataWarehouse^+^ (https://datawarehouse.dbd.go.th/). The Thai SME-GP is a platform that offers support measures for Thai SMEs in government procurement maintained by the Office of SMEs Promotion (OSMEP). The DBD DataWarehouse^+^ is a database for searching all juristic persons and businesses in Thailand maintained by the Department of Business Development (DBD), Ministry of Commerce.
2. For each company in the sample, we visited the company's website and examined whether the website published *a privacy policy* or *a cookie policy*. It is worth noting that some privacy criteria in the scoring model presented in Table 7 are relevant to a cookie policy, specifically criteria number 2, 5, 7, 12, 15, 16, 22, 23, 24, 25, and 30. This is because cookies can be considered a type of personal information. In other words, a cookie policy can be considered part of a privacy policy. Nonetheless, in practice, many companies have separate policies for cookies and privacy. In this particular case, we reviewed both policies together. Therefore, in this work, when referring to the assessment of a privacy policy, we practically assessed the cookie policy as part of it.
3. If an inspection of a company's website revealed that the website did not have a privacy and cookie policy, we would continue to investigate whether the website requested personal information from users. This could include a text box that prompts users to enter their phone number and contact information to apply for membership.
4. If a company's website had a privacy or cookie policy, we assessed the policy based on the scoring model described in Section 3.1. We carefully examined the privacy and cookie policies and assigned a score of zero (0) or one (1) for each of the 31 privacy criteria, depending on whether the policies failed to meet or met the scoring criteria outlined in Table A.15, respectively. Finally, we calculated each company's composite privacy policy score by the weighted sum of each criterion according to Equation (1). However, if a company did not provide a privacy or cookie policy on its website, a score of zero was assigned.

This study did not involve human subjects or their data; therefore, obtaining ethical approval was not applicable. The study relied exclusively on information freely available in the public domain, specifically website privacy policy and cookie policy pages that did not contain personal information. Nonetheless, ethical principles were observed throughout the study, including using legally and ethically obtained data.

### 3.3 Statistical data analyses of SME assessment results

This phase involves conducting various statistical analyses on the survey data obtained from SME assessments. The primary objectives of these analyses are as follows:

1. Analyzing the presence of personal data collection and a privacy policy on Thai SMEs' websites (refer to Section 4.1).
2. Assessing the PDPA-compliance situation of Thai SMEs based on the ten *privacy principles* of the scoring model (refer to Section 4.2).
3. Assessing the PDPA-compliance situation of Thai SMEs based on the 31 *privacy criteria* of the scoring model (refer to Section 4.3)
4. Analyzing the privacy policy scores and the differences in the privacy policy scores among groups of Thai SMEs, categorized by sector and TSIC section (refer to Section 4.4).

These statistical analyses provide valuable insights into the level of PDPA compliance among Thai SMEs and offer guidance for policy recommendations and support mechanisms to enhance compliance efforts. The findings shed light on the current state of PDPA compliance, identify areas where SMEs may need improvement, reveal compliance trends, and inform policy decisions and support strategies aimed at strengthening compliance efforts.

The subsequent section presents the results of statistical data analyses, including descriptive and inferential statistics. Sections 4.1, 4.2, 4.3, and 4.4 correspond to the four objectives mentioned above, respectively.

## 4 Results of statistical data analyses

### 4.1 Analysis of the presence of personal data collection and a privacy policy of SMEs

The purpose of this analysis is to study the overall characteristics of the samples regarding the presence of personal data collection and a privacy policy on Thai SMEs' websites. In particular, we aim to address the following questions:

1. What proportions of SMEs provided or did not provide privacy policies?
2. Among SMEs without privacy policies, what are the proportions of SMEs that collected or did not collect personal data?
3. How do these proportions vary across different sectors and TSIC sections?

The analysis results for the overall SMEs and the answers to the first and second questions are presented in Section 4.1.1. Sections 4.1.2 and 4.1.3 display the outcomes for SMEs categorized by sector and TSIC section, respectively, addressing the third question.

#### 4.1.1 Overall SMEs

**Figure 3:**
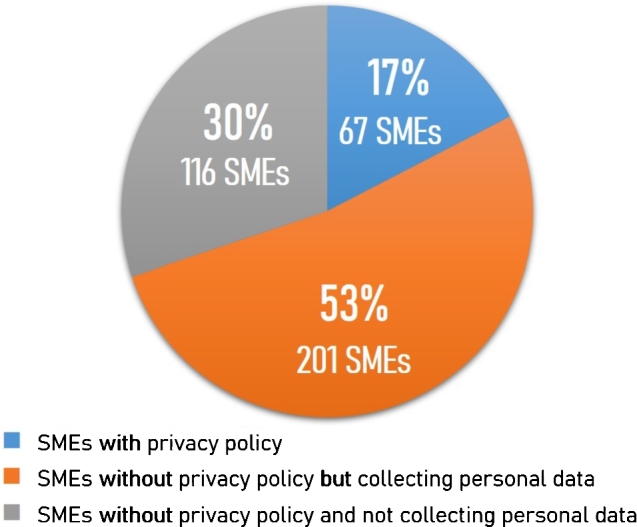
The proportion of SMEs based on the presence of personal data collection and a privacy policy.

For each SME in the sample, we visited the company's website and examined whether it published a privacy policy. In cases where a privacy policy was not provided, we examined whether the company collected personal data. Fig. 3 displays the proportion of SMEs based on the presence of personal data collection and a privacy policy. The results are categorized into three groups as follows:

1. *SMEs with privacy policy:* There were 67 SMEs that provided at least one of the following: a privacy policy, a cookie policy, or a cookie consent banner. This group accounted for only 17% of the sample.
2. *SMEs without privacy policy but collecting personal data:* There were 201 SMEs that did not have a privacy policy, a cookie policy, or a cookie consent banner, but they collected personal information from users. For example, the websites requested users to fill in personal information such as first name, last name, address, telephone number, and email address if users wanted to subscribe to services or required the websites to respond to their messages. This group of SMEs constituted 53% of the sample.
3. *SMEs without privacy policy and not collecting personal data:* There were 116 SMEs that did not provide any privacy policy, cookie policy, or cookie consent banner, and the websites did not collect personal information from users. This group formed 30% of the sample.

Strikingly, the results showed that the largest proportion (53%) of SMEs collected personal data from users without announcing a privacy policy regarding collecting, storing, and managing personal data on the websites to their users. Our result showed a slightly higher proportion than that reported by OSMEP [58], who surveyed Micro Small and Medium Enterprises (MSME) to assess their adaptation to Thailand's PDPA law in March 2022 using a questionnaire method. They found that 50.22% of MSMEs collected personal data from users but were unaware that PDPA would be enforced in Thailand.^4^

Compared to previous research in other countries, our findings indicate the most concerning situation regarding privacy policy compliance among Thai SMEs. Only 17% of Thai SMEs provided privacy policies, revealing the lowest level of compliance. In contrast, studies conducted in other regions demonstrate significantly higher compliance rates. Beldad et al. [13] found that 77% of Dutch municipal websites provided privacy policies. Similarly, Dias et al. [5] reported a provision rate of 26% for Portuguese municipal websites. Javed et al. [14], who examined South Asian popular websites, found that 63.8% of the sampled websites had privacy policies. Likewise, Reddick and Zheng [12] surveyed Chinese city government websites and found that 37% had privacy policies. These comparisons highlight the significant disparity between Thai SMEs and the compliance levels observed in other regions, emphasizing the urgent need for improved privacy policy compliance among Thai SMEs. The results suggested that most Thai SMEs were still unaware of PDPA, had a limited understanding of PDPA requirements, or were inaccessible to PDPA guidelines. Therefore, without government assistance or support in knowledge dissemination and establishment of mutual understanding, it might deprive Thai SMEs' opportunity to do business with foreign countries that have strict personal data protection measures.

#### 4.1.2 SMEs classified by sector

**Figure 4:**
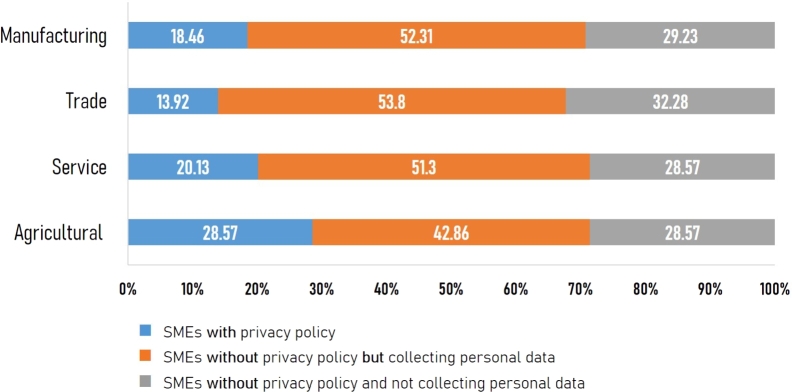
The proportion of SMEs in each sector based on the presence of personal data collection and a privacy policy.

In this section, we investigated the presence of personal data collection and a privacy policy of Thai SMEs at the sector level. Fig. 4 illustrates the proportion of SMEs in each sector based on the presence of personal data collection and a privacy policy.

The results in Fig. 4 consistently showed that SMEs without a privacy policy but collecting personal data constituted the most significant proportion in all sectors, while SMEs with a privacy policy formed the least significant proportion. Specifically, the proportion of SMEs without a privacy policy but collecting personal data in trade, manufacturing, service, and agricultural sectors were 53.8%, 52.31%, 51.3%, and 42.86%, respectively. The proportion of SMEs with a privacy policy in trade, manufacturing, service, and agricultural sectors were 13.92%, 18.46%, 20.13%, and 28.57%, respectively.

On one hand, it is expected that the trade sector, specifically motorcycle and auto retail, and the service sector, including tourism, hotels, beauty services, spa and massages, real estate, and freight activities, would collect the most personal data [58]. On the other hand, our survey revealed a worrying result that more than half of the entrepreneurs in these sectors (53.8% of the trade sector and 51.3% of the service sector) were still not ready for PDPA and did not announce a privacy policy for their customers. Hence, it is vital that these sectors get adequate knowledge and PDPA compliance preparation to cope with the increase in personal data collection and potential data leakage.

#### 4.1.3 SMEs classified by TSIC section

**Figure 5:**
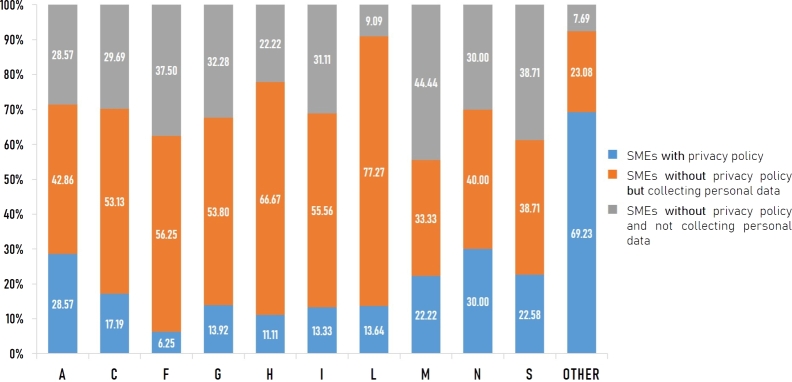
The proportion of SMEs in each TSIC section based on the presence of personal data collection and a privacy policy.

Here, we conducted a more detailed analysis of the presence of personal data collection and a privacy policy of Thai SMEs at the TSIC section level. Fig. 5 shows the proportion of SMEs in each TSIC section based on the presence of personal data collection and a privacy policy.

Our survey found that in all TSIC sections except sections M and *Other*, the proportions of SMEs without a privacy policy but collecting personal data were the largest. In particular, the real estate activities (section L) and transportation and storage (section H) were the most concerning sections because the proportions of SMEs without a privacy policy but collecting personal data in these sections were as high as 77.27% and 66.67%, respectively. In addition, both were among business activities that [58] expected to collect the most personal information. Thus, policymakers and government authorities should primarily support these business activities by providing knowledge and guidelines, offering specialist advice, and building networks to raise awareness and facilitate the sharing of knowledge and experiences in implementing the PDPA.

On the other hand, section *Other* was the strongest business group, evident from the highest proportion of SMEs with a privacy policy at 69.23%. As mentioned earlier in Section 3.2.2, the section *Other* comprised a compilation of TSIC sections with small proportions of SMEs, each accounting for less than 1.14% of all Thai SMEs. Examples of these sections included mining and quarrying (section B), electricity supply (section D), water supply (section E), financial and insurance (section K), and international organizations (section U). It is worth noting that SMEs in these sections possess the capability to adapt to Thailand's PDPA, engage in specialized economic activities, and rely on advanced expertise.

Regarding SMEs without a privacy policy and not collecting personal data, the professional, scientific, and technical activities (section M) showed the highest proportion of SMEs in this category. SMEs in this section probably only provided company information or announcement on their websites without collecting personal data. Nevertheless, this remained a concern as the absence of a privacy or cookie policy represented a low level of awareness and preparedness for PDPA.

### 4.2 Analysis of the ten privacy principles of SMEs

This analysis seeks to understand the degree to which Thai SMEs comply with each of the ten *privacy principles* presented in Table 7. In particular, we are interested in answering the question: What are the *privacy principles* that Thai SMEs comply with the most and the least? Based on the results in Section 4.1.1, where 67 SMEs in the samples provided a privacy policy on their websites, in this section, we further examined this group of SMEs on the average score of each *privacy principle*.

**Figure 6:**
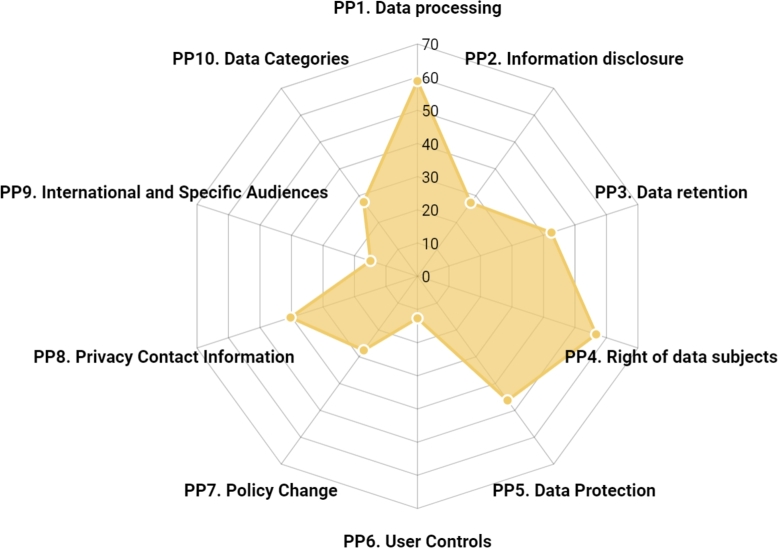
The average score of the ten privacy principles of SMEs providing a privacy policy.

For each SME, the score of a privacy principle (PP) was computed similarly to Equation (1). However, instead of computing across all 31 *privacy criteria*, here we computed only *privacy criteria* within a PP of consideration, then normalized the total score to 100. Afterward, the PP scores of the 67 SMEs were averaged and plotted as a radar chart shown in Fig. 6.

Concerning the ten privacy principles, the analysis focused on the 67 SMEs that provided a privacy policy. The results revealed that *PP1. Data processing* had the highest score of 58.81, followed by *PP4. Rights of data subjects* with a score of 56.72, and *PP5. Data protection* with a score of 46.27. These results indicate that among SMEs who disclosed a privacy policy, they demonstrated relatively strong compliance with the PDPA in terms of informing users about the meaning of personal data, the purpose of data collection, data subjects' rights, and security measures.

Contrastingly, the least compliant privacy principle was *PP6. User controls* with a score of 12.69. This finding indicates that, despite having a privacy policy, most SMEs did not provide user controls or opt-out options for their data, including not providing a consent form for personal data collection, not facilitating data subjects to request withdrawal of consent, or not providing cookie setting options that allowed users to reject non-necessary cookies collection. These shortcomings may be attributed to the absence of clear guidelines from government authorities regarding these privacy principles for SMEs, as well as budget constraints that hinder the implementation of necessary systems due to high development costs. Additionally, SME entrepreneurs may need to seek advice from PDPA specialists and website developers to incorporate appropriate technologies that cater to the needs of both data subjects and business activities.

### 4.3 Analysis of the 31 privacy criteria of SMEs

This analysis aims to study the degree to which Thai SMEs comply with each of the 31 *privacy criteria* presented in Table 7. Here, our study seeks to address the following questions:

1. What are the percentages of SMEs that comply with each *privacy criterion* in the scoring model?
2. Which *privacy criteria* do Thai SMEs comply with the most and the least?
3. How is the distribution of the number of privacy criteria complied with by SMEs? Additionally, what are the minimum, maximum, and average number of privacy criteria complied with by SMEs?

**Figure 7:**
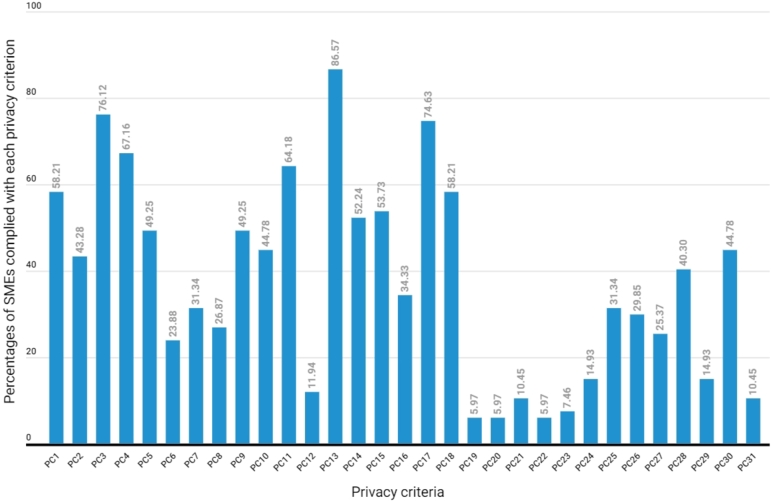
The percentages of SMEs complied with each of the 31 privacy criteria (PC).

Like the previous section, here we focus our study on the 67 SMEs that published a privacy policy on their websites. We examine the proportion of those SMEs fulfilling the 31 *privacy criteria* of the scoring model. Fig. 7 shows the percentages of SMEs that complied with each of the 31 *privacy criteria (PC)*.

Fig. 7 indicates that the privacy criterion that SMEs complied with the most was *PC13. Accessibility*. Among the SMEs providing a privacy policy, 86.57% complied with PDPA fundamentals regarding website accessibility by designing a website for easy visibility and accessibility to a privacy policy, such as providing clear text or a pop-up banner linked to a privacy policy.

The second most compliant privacy criterion by SMEs was *PC3. Purpose of personal data collection*. In total, 76.12% of SMEs announcing a privacy notice were aware of the role of the data controller and that it is necessary to inform data subjects about the purpose of collection, usage, and disclosure of personal data.

The next most compliant privacy criterion among SMEs was *PC17. Hypertext Transfer Protocol Secure (HTTPS)*. About three-fourths (74.63%) of the SMEs that provided a privacy notice implemented data encryption or SSL protocol in the form of HTTPS to ensure the secure transmission of information between users' computers and the website.

On the contrary, three privacy criteria were the least compliant by SMEs, namely, *PC19. Statement notifying users of the limitation or scope of its liability when visiting other websites*, *PC20. Consent form*, and *PC22. Non-necessary cookie rejection*. Only about six percent (5.97%) of SMEs complied with these privacy criteria, indicating a concern that most SMEs rarely notify data subjects of their limitation or scope of liability when data subjects click on links to other websites from their websites. Furthermore, most SMEs seldom provided a consent form for personal data collection and an option for data subjects to reject non-necessary cookies. These issues may arise from an unawareness of proper practices and unclear PDPA compliance guidelines.

Additionally, there are two other critical issues that SMEs should inform data subjects about; failure to do so may result in fines of up to 1 million Thai baht (approximately 30,000 USD) according to PDPA Sections 23 and 82. These issues relate to *PC10. Storage and data retention period* and *PC28. Contact information* of the data controller or the responsible person (Data Protection Officer: DPO). However, it was found from the survey that only 44.78% and 40.30% of SMEs provided information about the data retention period and the contact information in their privacy policies, respectively.

**Figure 8:**
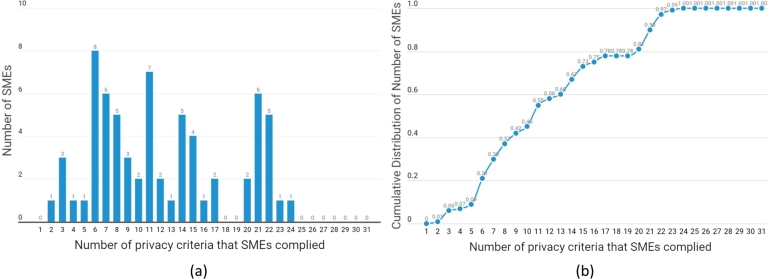
(a) A histogram of the number of privacy criteria fulfilled by SMEs, (b) A cumulative distribution of the number of privacy criteria fulfilled by SMEs.

Next, we are interested in studying the frequency distribution of the number of privacy criteria complied with by SMEs. Figs. 8(a) and 8(b) represent a histogram and a cumulative distribution, respectively, of the number of privacy criteria that SMEs complied with. The Y-axis in Fig. 8(a) shows the number of SMEs that fulfilled a specific number of privacy criteria displayed on the X-axis.

It can be seen from Fig. 8(a) that the number of privacy criteria fulfilled by SMEs was a multimodal distribution, with the highest and lowest numbers of compiled privacy criteria at 24 and 2, respectively. In particular, none of the SMEs in the sample managed to comply with up to 80% of the 31 privacy criteria (i.e., 25 privacy criteria). On average, SMEs announcing a privacy policy complied with only 12.15 out of 31 privacy criteria. The cumulative distribution in Fig. 8(b) indicates that about half of SMEs only complied with fewer than 11 out of 31 privacy criteria. Furthermore, about three-fourths of the SMEs complied with fewer than 16 of 31 privacy criteria.

The above survey results revealed a concerning situation of PDPA compliance among Thai SMEs. The low compliance observed may be attributed to SMEs' limited knowledge and understanding of PDPA guidelines, as well as a lack of awareness regarding the essential information that should be communicated to data subjects via privacy policies. It is also possible that most SMEs lack specific PDPA guidelines regarding privacy policies. Therefore, this paper aims to provide a practical and straightforward scoring model with a privacy criteria checklist. This model will enable SMEs to assess the compliance of their privacy policies with the PDPA and identify areas for improvement in terms of PDPA compliance. Furthermore, policymakers and government authorities should focus on providing knowledge and guidelines, offering specialist advice, and building networks to raise awareness and facilitate knowledge sharing among communities. Priority should be given to addressing the lowest compliance rates in privacy criteria, namely *PC19, PC20,* and *PC22*.

### 4.4 Analysis of the privacy policy scores and the differences in the privacy policy scores among groups of SMEs

This analysis aims to examine the privacy policy scores and the differences in these scores among groups of SMEs, categorized by sector and TSIC section. The privacy policy scores were computed for each SME based on the scoring model according to Equation (1). The total score for each SME is 100. In case an SME did not provide a privacy policy, a zero score was given. These privacy policy scores would provide insights into the level of compliance with privacy principles and criteria outlined in the scoring model.

Furthermore, the analysis aims to identify variations in privacy policy scores across different sectors and TSIC sections. By examining these variations, valuable insights can be gained regarding the level of privacy policy compliance across sectors and TSIC sections. This analysis allows for identifying significant differences or patterns in privacy policy scores among sectors and TSIC sections. By comparing the privacy policy scores, we can identify sectors or TSIC sections that demonstrate higher or lower levels of compliance. This information is crucial for understanding the overall compliance landscape and developing targeted strategies to improve privacy policy practices in specific sectors or sections.

Descriptive and inferential statistical data analyses of the privacy policy scores are presented in the subsequent sections, respectively.

#### 4.4.1 Descriptive statistical analysis

Table 10 presents the descriptive statistics of the privacy policy scores for all SMEs included in the sample. The average privacy policy score obtained was 6.1909 out of 100, with a standard deviation of 16.04091. The minimum score recorded was 0, indicating the lowest level of compliance, while the maximum score achieved was 78, representing the highest level of compliance among the SMEs. These statistics shed light on the overall state of privacy policy practices among Thai SMEs, indicating significant room for improvement. The average score of 6.1909 out of 100 reflects a very low level of compliance with privacy policies. This finding highlights the need for attention and efforts to enhance privacy protection practices among Thai SMEs. It suggests substantial gaps and deficiencies in implementing privacy policies, potentially exposing personal data to risks and impacting individuals' privacy rights.

**Table 10:** The descriptive statistics of the composite privacy policy scores of all SMEs in the sample.

	N	Average	Standard deviation	Minimum	Maximum
All SMEs	384	6.1909	16.0409	0	78.00

The wide range between the minimum and maximum scores also indicates a significant variation in privacy policy compliance among SMEs. Some SMEs have demonstrated a higher level of compliance with privacy policies, while others have fallen considerably short. This variation emphasizes the importance of identifying the aspects contributing to lower scores and implementing targeted strategies to address them.

Table 11 displays the descriptive statistics of the privacy policy scores of SME groups categorized by sector. The findings revealed a consistent and challenging PDPA compliance situation among Thai SMEs across all sectors. The average scores ranged from 4.4937 to 6.6460 out of 100, indicating a low level of compliance with privacy policies in every sector. The similarity in average scores suggests that factors impacting privacy policy compliance are widespread and not specific to a particular sector.

**Table 11:** The descriptive statistics of the composite privacy policy scores of SME groups according to sectors.

Sector	N	Average	Standard deviation	Minimum	Maximum
Manufacturing	65	4.4937	13.4306	0	75.83
Trade	158	6.6460	16.8137	0	73.83
Service	154	5.8086	18.1518	0	78.00
Agricultural	7	6.1909	12.0999	0	32.33

Furthermore, the analysis of standard deviation and maximum scores provides additional insights into the compliance levels within sectors. The agricultural sector, in particular, demonstrated consistently low levels of PDPA compliance, as indicated by a low standard deviation of 12.0999 and a maximum score of 32.33. This suggests that SMEs in the agricultural sector face significant challenges in implementing and adhering to privacy policies, resulting in lower overall compliance scores.

Table 12 displays that the average privacy policy scores of SMEs classified by TSIC section varied from 1.3750 to 26.3346 out of 100, reflecting a large variability of the PDPA compliance level among different TSIC sections.

**Table 12:** The descriptive statistics of the composite privacy policy scores of SME groups according to TSIC sections.

TSIC Section	N	Average	Standard deviation	Minimum	Maximum
A	7	5.8086	12.0999	0	32.33
C	64	5.7264	15.2101	0	73.83
F	16	1.3750	5.5000	0	22.00
G	158	4.4937	13.4306	0	75.83
H	9	2.5744	7.7233	0	23.17
I	45	5.2702	16.5913	0	78.00
L	22	5.4700	16.2398	0	61.67
M	9	15.2033	30.1862	0	70.50
N	10	6.1330	10.4821	0	29.00
S	31	10.2255	21.4231	0	65.83
*Other*	13	26.3346	23.5049	0	65.50

Considering these findings, it is essential for policymakers and government authorities to prioritize support for businesses operating in TSIC sections with lower compliance levels. Specifically, attention should be given to SMEs in construction (section F) and transportation and storage (section H). These sections demonstrated the lowest average privacy policy scores (1.3750 and 2.5744), as well as the lowest standard deviations (5.5000 and 7.7233) and maximum scores (22.00 and 23.17). These consistent low scores across all SMEs within these TSIC sections highlight the need for targeted interventions and assistance to improve PDPA compliance. By focusing efforts on TSIC sections with lower compliance levels, policymakers can address the specific challenges faced by SMEs in the construction (section F) and transportation and storage (section H). This approach would help elevate the overall compliance landscape and contribute to enhancing privacy policy practices among Thai SMEs.

#### 4.4.2 Inferential statistical analysis

The purpose of this section is to test the hypotheses for the differences in the privacy policy scores among groups of SMEs, where groups are determined by sector and TSIC section. Analyzing these differences provides valuable insights into the extent of privacy policy compliance across sectors and TSIC sections. This analysis allows for identifying significant differences or patterns in privacy policy scores among sectors and TSIC sections. Such information is crucial for policymakers and government agencies in developing targeted strategies to improve privacy policy practices in specific sectors or TSIC sections.

We utilized the Kruskal-Wallis test with post hoc analysis to identify differences among various groups of SMEs. The Kruskal-Wallis is a non-parametric test considered an alternative to one-way ANOVA and is conducted to determine whether there are any differences among more than two groups where the data is not normally distributed [19], [20], [21], [22], [23]. Subsequently, a post hoc analysis was performed to determine the groups with significant differences for the variables that had significant differences among groups [59], [60], [61], [23], [62]. For a post hoc analysis, we applied Dunn's test, an appropriate non-parametric pairwise multiple comparison procedure when a Kruskal-Wallis test is rejected. Dunn's test is recommended when sample sizes among groups are unequal, or in the presence of tied ranks [24], [21], [25]. For all the following statistical data analyses, the significance level was set at 0.05.

**(1) Analysis of the differences in the composite privacy policy scores among sectors**

This analysis aims to identify significant differences in privacy policy scores among sectors. The hypotheses of the Kruskal-Wallis test for the differences in the composite privacy policy scores among sectors are given below.

$H_{0}$: *There is no difference among sectors for the composite privacy policy scores.*

$H_{1}$: *At least one of the sectors differs from the other sectors for the composite privacy policy scores.*

The Kruskal-Wallis test was conducted to examine the differences in the composite privacy policy scores according to sectors of SMEs. No significant differences, $\chi^{2}(3)=2.921,p=0.404$, were found among the four sectors of SMEs (manufacturing, trade, service, and agricultural sectors) for the composite privacy policy scores. This finding, supported by the result presented in Table 11, suggests that Thai SMEs in various sectors face similar difficulties in achieving PDPA compliance. Regardless of the sector they operate in, SMEs struggle to meet the requirements and guidelines set forth by the PDPA.

**(2) Analysis of the differences in the composite privacy policy scores among TSIC sections**

This analysis aims to identify significant differences in privacy policy scores among TSIC sections. The hypotheses of the Kruskal-Wallis test for the differences in the composite privacy policy scores among TSIC sections are given below.

$H_{0}$: *There is no difference among TSIC sections for the composite privacy policy scores.*

$H_{1}$: *At least one of the TSIC sections differs from the other TSIC sections for the composite privacy policy scores.*

The Kruskal-Wallis test showed a statistically significant difference in the composite privacy policy scores among TSIC sections of SMEs, $\chi^{2}(10)=31.285,p=0.001$. In other words, there was a significant difference in at least one of the TSIC sections for the composite privacy policy scores. Dunn's pairwise post hoc test was then performed for the TSIC section to determine the groups with significant differences. The results of Dunn's test revealed that there were significant differences (p<0.05) between section *Other* and all the remaining TSIC sections. In particular, all the TSIC sections (except section *Other*) showed significantly lower composite privacy policy scores than the section *Other*.

This result shows that categorizing SMEs based on their economic activities (TSIC sections) influences the PDPA compliance level. Section *Other*, which comprises specialized and advanced economic activities, including mining and quarrying, electricity supply, water supply, financial and insurance, and international organizations, had significantly higher composite privacy policy scores than the remaining TSIC sections (sections A, C, F, G, H, I, L, M, N, and S). This finding reflects the section's capability to comply with the new Thailand's PDPA law. Therefore, policymakers and government authorities should raise awareness of PDPA by categorizing SMEs based on TSIC sections and implementing tailored policies for each group.

Next, we further investigated the differences in the *privacy principle* (PP) scores for each of the ten *privacy principles* among different groups of TSIC sections.

**(3) Analysis of the differences in the *privacy principle* (PP) scores among TSIC sections**

In this analysis, we focused on analyzing the differences in the scores of the *privacy principles* (PPs) among TSIC sections. To compute the PP scores for each SME, we followed a similar calculation approach as described in Equation (1). However, instead of computing across all 31 *privacy criteria*, here we computed only *privacy criteria* within the respective PP of interest. These scores were then normalized to a scale of 0 to 100. In case an SME did not provide a privacy policy on its website, a zero score was given to all PPs. Afterward, the PP scores of all SMEs were used for hypothesis testing of the differences among TSIC sections by the Kruskal-Wallis test. This test allowed us to determine if there were statistically significant variations in the PP scores across different TSIC sections.

For each *i* of the ten *privacy principles* (PP), the hypotheses of the Kruskal-Wallis test for the differences among TSIC sections are given below.

$H_{0}$: *There is no difference among TSIC sections for the privacy principle i (PPi) scores.*

$H_{1}$: *At least one of the TSIC sections differs from the other TSIC sections for the privacy principle i (PPi) scores.*

Table 13 shows the results of the Kruskal-Wallis test for the differences in the ten privacy principle (PP) scores among TSIC sections. No significant differences were found among TSIC sections for the *PP6. User controls*, *PP7. Policy Change*, *PP9. International and specific audiences*, and *PP10. Data categories* scores.

**Table 13:** The Kruskal-Wallis test for the differences in the ten privacy principle (PP) scores among TSIC sections.

PP score	*χ*^2^ (df=10)	p-value*	Conclusion
PP1	31.056	**0.001**	Significant different among TSIC sections
PP2	21.761	**0.016**	Significant different among TSIC sections
PP3	29.321	**0.001**	Significant different among TSIC sections
PP4	33.594	**0.000**	Significant different among TSIC sections
PP5	42.686	**0.000**	Significant different among TSIC sections
PP6	15.601	0.112	No significant different among TSIC sections
PP7	12.928	0.228	No significant different among TSIC sections
PP8	30.095	**0.001**	Significant different among TSIC sections
PP9	5.852	0.828	No significant different among TSIC sections
PP10	18.076	0.054	No significant different among TSIC sections

* Values in bold are significantly different among TSIC sections at 5% level (Kruskal-Wallis test; p<0.05).

On the contrary, the Kruskal-Wallis test showed a statistically significant difference in the *PP1. Data processing*, *PP2. Information disclosure*, *PP3. Data retention*, *PP4. Right of data subjects*, *PP5. Data protection*, and *PP8. Privacy contact information* scores among TSIC sections of SMEs. In other words, there was a significant difference in at least one of the TSIC sections for each of these PP scores. To further investigate these significant differences, Dunn's pairwise post hoc test was performed. The results of this test determined the specific TSIC sections with significant differences for these PP scores with significant differences (*PP1, PP2, PP3, PP4, PP5, PP8*). Table 14 displays the results of Dunn's pairwise comparison post hoc test.

**Table 14:** The Dunn's pairwise comparison post hoc test for the differences in the ten privacy principle (PP) scores among TSIC sections.

PP score	Dunn's pairwise comparison post hoc test†
PP1	A, C, F, G, H, I, L, M, N, S vs. *Other*
PP2	A, C, F, G, H, I, L, N, S vs. *Other*
PP3	A, C, F, G, H, I, L, M, N, S vs. *Other*; I vs. S; G vs. S
PP4	A, C, F, G, H, I, L, M, N, S vs. *Other*
PP5	A, C, F, G, H, I, L, M, N, S vs. *Other*
PP6	-
PP7	-
PP8	A, C, F, G, H, I, L, N, S vs. *Other*; G vs. M
PP9	-
PP10	-

† Show only TSIC sections with significant differences (Dunn's test; p<0.05).

For *PP1. Data processing*, *PP4. Right of data subjects*, and *PP5. Data protection*, the Dunn's test showed that there were significant differences (p<0.05) between the section *Other* and all the remaining TSIC sections. In particular, all the TSIC sections, except section *Other*, exhibited significantly lower scores for *PP1*, *PP4*, and *PP5* compared to section *Other*.

For *PP2. Information disclosure*, the Dunn's test showed that there were significant differences (p<0.05) between the section *Other* and sections A, C, F, G, H, I, L, N, and S. Particularly, sections A, C, F, G, H, I, L, N, and S showed significantly lower *PP2* scores compared to section *Other*.

For *PP3. Data retention*, the Dunn's test indicated significant differences (p<0.05) in three scenarios: (1) between the section *Other* and all the remaining TSIC sections, (2) between accommodation and food service activities (section I) and other service activities (section S), and (3) between wholesale and retail trade (section G) and other service activities (section S). Specifically, section *Other* showed significantly higher *PP3* scores than all the remaining TSIC sections, section S showed significantly higher *PP3* scores than sections G and I.

For *PP8. Privacy contact information*, the Dunn's test revealed significant differences (p<0.05) in two cases (1) between section *Other* and sections A, C, F, G, H, I, L, N, and S, and (2) between wholesale and retail trade (section G) and professional, scientific, and technical activities (section M). In particular, section *Other* showed significantly higher *PP8* scores compared to sections A, C, F, G, H, I, L, N, and S, while section M exhibited significantly higher *PP8* scores than section G.

Based on the above analysis of each privacy principle, it was observed that there were no significant differences in the scores of *PP6. User controls*, *PP7. Policy Change*, *PP9. International and specific audiences*, and *PP10. Data categories* among TSIC sections. This implies that the compliance rates for these privacy principles are relatively consistent across different TSIC sections. Consequently, policymakers and government authorities can implement universal policy approaches to improve compliance with these particular privacy principles across various TSIC sections.

On the other hand, it is evident that the PDPA compliance levels for *PP1. Data processing*, *PP2. Information disclosure*, *PP3. Data retention*, *PP4. Right of data subjects*, *PP5. Data protection*, and *PP8. Privacy contact information* differed among TSIC sections. In light of these findings, policymakers and government authorities should adopt tailored approaches to enhance PDPA awareness and compliance within each TSIC section.

One possible strategy is prioritizing assistance and support for SMEs operating in vulnerable TSIC sections. These sections may require additional guidance and resources to improve their compliance. By focusing on providing targeted support to these sections, policymakers can help them learn from the exemplary practices of other TSIC sections and facilitate their progress toward better PDPA compliance.

Another approach involves leveraging SMEs in strong TSIC sections as role models for those in other TSIC sections. For instance, in the case of *PP3. Data retention*, the exemplary practices of section *Other* can serve as a valuable reference for all the remaining TSIC sections. Similarly, section S can act as a role model for sections G and I, demonstrating effective strategies in achieving PDPA compliance in *PP3. Data retention*.

Furthermore, the concept of leveraging SMEs in strong TSIC sections as role models can also be extended to other privacy principles. Taking *PP8. Privacy contact information* as an example, section *Other* can serve as a role model for sections A, C, F, G, H, I, L, N, and S in terms of effectively providing contact information of the data controller or the Data Protection Officer (DPO). By examining the practices of section *Other*, these TSIC sections can gain insights into establishing clear and accessible channels for data subjects to communicate their privacy concerns and inquiries to the data controller.

Similarly, section M can serve as a role model for section G in achieving compliance with *PP8. Privacy contact information*. By studying the successful approaches implemented by section M, section G can enhance its practices in ensuring that appropriate contact information is readily available to data subjects for privacy-related matters.

## 5 Recommendations

### 5.1 Recommendations for SMEs

Our analysis provided evidence that the overall readiness of SMEs for Thailand's PDPA law was very challenging. Based on our findings, SMEs collecting personal data from users but not providing a privacy policy regarding collecting, storing, and managing personal data constituted the largest proportion. In contrast, SMEs with a privacy policy formed the smallest proportion. Therefore, it is essential for Thailand's SMEs to raise their digital skills and acknowledge the significance of complying with the personal data protection law in order to thrive in the digital economy.

Our *privacy policy scoring model* can serve as a practical checklist for SMEs to self-assess the compliance of their privacy policies with the PDPA and identify areas for improvement in terms of privacy principles and criteria. Based on our survey results, which are classified by sector and TSIC section, SMEs can also benchmark themselves against others in the same sector or TSIC section.

According to our survey results in Section 4.2, the least compliant privacy principle among SMEs was *PP6. User controls*. SMEs should be aware that to comply with PDPA, they are not only required to announce a privacy policy to their users, but they are also needed to provide user controls and opt-out options on users' personal data, which includes providing a consent form for personal data collection, facilitating data subjects' withdrawal of consent, and providing cookie setting options that allow users to reject non-necessary cookies collection.

Section 4.3 showed that *PC19. Statement notifying users of the limitation or scope of its liability when visiting other websites*, *PC20. Consent form*, and *PC22. Non-necessary cookie rejection* were the three least compliant privacy criteria among SMEs. In addition to implementing a consent form for personal data collection and providing an option for data subjects to reject non-necessary cookies, SMEs should also ensure that they notify data subjects about the limitation or scope of their liability if data subjects access other websites through their own websites.

Last but not least, Thai SMEs should be aware that the PDPA Sections 23 and 82 require data controllers to inform data subjects regarding two other critical pieces of information: *PC10. Storage and data retention period* and *PC28. Contact information* of the data controller or the responsible person (Data Protection Officer: DPO). Violation of such PDPA sections may lead to fines of up to 1 million Thai baht (approximately 30,000 USD).

### 5.2 Policy recommendations for policymakers and government authorities

Governments play a vital role in effectively enforcing personal data protection laws [63], [64]. This paper proposed a scoring model for assessing PDPA-compliant privacy policies. Policymakers and government authorities can utilize this model to evaluate businesses in Thailand, enabling them to gain a comprehensive understanding of the PDPA compliance situation and make informed decisions to promote awareness of personal data protection to the right target group. The paper also demonstrated the application of the scoring model to Thai SMEs, key economic drivers of Thailand, to gain insights into the PDPA compliance situation within the SME sector.

According to the analysis conducted in Section 4.3, it is crucial for policymakers and government authorities to provide Thai SMEs with comprehensible PDPA information, access to experts consultation, and opportunities to engage in communities that promote awareness and experience sharing regarding PDPA implementation. These efforts should especially focus on addressing the least-compliant privacy criteria first, namely, *PC19. Statement notifying users of the limitation or scope of its liability when visiting other websites*, *PC20. Consent form*, and *PC22. Non-necessary cookie rejection*.

Section 4.4.1 showed a large variability of the PDPA compliance level among different TSIC sections. Therefore, it is imperative for the government to prioritize support for businesses operating in TSIC sections with low compliance levels, especially SMEs in the construction sector (section F) and the transportation and storage sector (section H).

Results in Section 4.4.2 indicated that the PDPA compliance levels of the *PP1. Data processing*, *PP2. Information disclosure*, *PP3. Data retention*, *PP4. Right of data subjects*, *PP5. Data protection*, and *PP8. Privacy contact information* differed among TSIC sections. Therefore, the government should adopt approaches tailored to the specific requirements of each TSIC section. These approaches may involve providing relevant content, skills, and expert guidance to address the unique challenges faced by each TSIC section. Additionally, the government should consider prioritizing assistance for SMEs in vulnerable TSIC sections or leveraging SMEs in strong TSIC sections as role models for their counterparts in other sections, while promoting knowledge exchange and networking to strengthen the overall compliance landscape.

## 6 Limitations and future directions

While our proposed privacy policy scoring model offers several benefits for decision-making, it is crucial to acknowledge and address its limitations. The following limitations should be taken into consideration:

### 6.1 Reliance on equal weights approach

This work used the equal weights approach to assign equal importance to all the ten privacy principles in the scoring model. Although this approach provides simplicity and ease of implementation and is a common technique in calculating a composite index from multi-criteria in social sciences [55], [56], it may not reflect the actual preferences and priorities of decision-makers. Future research should explore more rigorous methods from Multi-Criteria Decision Making (MCDM) [17], [18] for criterion weighting, such as the Analytic Hierarchy Process (AHP) [52], [53] or the Technique for Order of Preference by Similarity to Ideal Solution (TOPSIS) [54], to derive more accurate and representative weights for each criterion.

### 6.2 Subjectivity in scoring criteria

Although we conducted a thorough content analysis [48] of laws, guidelines, and academic research to select and define the scoring criteria carefully, it is essential to recognize that these criteria may still involve a level of subjectivity. This subjectivity can introduce bias and influence the reliability and objectivity of the model's results. To mitigate this limitation, future research should focus on refining and standardizing the scoring criteria to minimize subjectivity. One approach is to employ a consensus-based method involving multiple evaluators or PDPA experts, such as the Delphi method [65]. This would involve iterative rounds of anonymous feedback and discussion to converge toward a collective agreement on scoring assignments. Additionally, calibration through providing evaluators with examples or training cases with known scores can help align their judgments and ratings. Forming expert panels or committees composed of PDPA experts can also minimize subjectivity. Through collective discussions, consensus-building, and knowledge sharing, experts can reach agreement on scoring assignments based on their collective expertise.

### 6.3 Limited external validation

Although our privacy policy scoring model was developed based on a comprehensive literature review, it is important to recognize the absence of external validation. To ensure the model's reliability and generalizability, further validation is needed through external expert evaluations or sensitivity analysis. Future research should focus on conducting external validation studies involving independent experts or organizations to ensure the model's reliability and robustness. These experts can critically review the scoring model, criteria weights, and the overall decision-making process. Their judgment can also be used to compare the model's results with their assessments, providing insights into the model's accuracy. Additionally, performing sensitivity analysis is another effective approach to verify the robustness and stability of the scoring model [17]. Sensitivity analysis involves systematically varying the privacy criteria weights or scoring assignments to examine their impact on the overall results. By examining how changes in weights or scores affect the outcomes, we can gain a deeper understanding of the model's performance.

### 6.4 Time-specific evaluation

Our SMEs assessment utilized the proposed scoring model, but it is important to recognize the limitation of temporal relevance. The survey was conducted in December 2021, examining their readiness for PDPA six months before the law's full enforcement on June 1st, 2022. Since the time of assessment, the business landscape and context may have evolved. To address this limitation, we recommend conducting a follow-up study using the proposed scoring model in the future. This will provide valuable insights into the model's applicability in the current business environment, allowing for more up-to-date and relevant decision-making. Furthermore, it will enable a comparison of privacy policy scores before and after the promulgation of the law.

## 7 Conclusions

Through a content analysis of laws, guidelines, and academic research, we developed a multi-criteria *privacy policy scoring model* consisting of 10 privacy principles and 31 privacy criteria to assess PDPA-compliant privacy policies. In the survey conducted on 384 SME websites using stratified random sampling, the presence of personal data collection and privacy policies was assessed. The findings revealed a poor overall PDPA compliance situation among Thai SMEs, with an average score of 6.1909 out of 100. Over half of them (53%) collected personal data without announcing privacy policies, while only 17% provided privacy policies. On average, SMEs with privacy policies complied with only 12.15 out of 31 privacy criteria. There were three privacy criteria with the lowest compliance rate of 5.97%: *PC19. Statement notifying users of the limitation or scope of its liability when visiting other websites*, *PC20. Consent form*, and *PC22. Non-necessary cookie rejection*.

In the analysis of the ten privacy principles, the survey found that the least compliant privacy principle among SMEs was *PP6. User controls* with a score of 12.69 out of 100. This indicates that most SMEs did not provide an opt-out option for users on their data, failed to offer a consent form for personal data collection, did not facilitate data subjects to request consent withdrawal, and did not allow users to reject non-necessary cookies.

Through the Kruskal-Wallis test and Dunn's post hoc analysis, we studied the differences in privacy policy scores among different sectors and TSIC sections of Thai SMEs. The statistical analyses showed a significant difference in privacy policy scores among TSIC sections. Specifically, all TSIC sections showed significantly lower privacy policy scores than the section *Other*, which comprises specialized and advanced economic activities such as mining, electricity supply, water supply, financial and insurance, and international organizations. Furthermore, when considering ten privacy principle (PP) scores, TSIC sections performed differently in *PP1. Data processing*, *PP2. Information disclosure*, *PP3. Data retention*, *PP4. Right of data subjects*, *PP5. Data protection*, and *PP8. Privacy contact information*. As a policy implication, this outcome highlights the necessity for policymakers to adopt different strategies to address the specific challenges of each TSIC section.

In conclusion, the proposed scoring model provides a structured and quantitative framework for evaluating privacy policies, enabling businesses and policymakers to gain insights into the current level of PDPA compliance. Businesses can utilize the model to identify gaps for improvement in their privacy policies, benchmark themselves with peers in the same sector, and ensure alignment with legal requirements and best practices. Policymakers and government authorities can employ the model to evaluate businesses. This enables them to understand the compliance landscape and formulate targeted policies tailored to specific sectors to improve compliance efforts. These customized policies may involve prioritizing support for vulnerable sectors or leveraging businesses in strong sectors as examples for other sectors to follow. By adopting the model as a standardized tool for regular assessment, stakeholders can track compliance progress over time, potentially leading to an overall improvement in privacy policy practices.

## CRediT authorship contribution statement

**Panchapawn Chatsuwan, Tanawat Phromma:** Performed the experiments; Analyzed and interpreted the data; Contributed reagents, materials, analysis tools or data; Wrote the paper. **Navaporn Surasvadi:** Conceived and designed the experiments; Analyzed and interpreted the data; Contributed reagents, materials, analysis tools or data; Wrote the paper. **Suttipong Thajchayapong:** Analyzed and interpreted the data; Contributed reagents, materials, analysis tools or data; Wrote the paper.

## Declaration of generative AI and AI-assisted technologies in the writing process

During the preparation of this work, the authors used ChatGPT to assist in improving the language and readability of the manuscript. After the AI-assisted language editing process, the authors thoroughly reviewed and edited the content to ensure its accuracy. The authors take full responsibility for the content of the publication and affirm that the AI tool was employed solely to aid in the language editing process.

## Declaration of Competing Interest

The authors declare that they have no known competing financial interests or personal relationships that could have appeared to influence the work reported in this paper.

## Figures and Tables

**Table A.15:** Scoring criteria.

**1. Data processing (Collection, Purpose)**		**References**

(1) Give meaning of personal data	1 = The data controller explained the meaning of personal data according to the PDPA scope for personal data classification.	Article 37 in [27]
	0 = The data controller did not explain the meaning of personal data or give a personal data definition under the PDPA scope.	

(2) Give meaning of cookies	1 = The data controller explained or mentioned the definition of a cookie as a small text file stored in the browser or hard disk of the user's computer, such as personal data registered with the website while accessing website information. The cookie file that is automatically created when users visit websites from which the data controller can collect personal data is shown in the privacy policy/banner/pop-up.	[50]; [51] Ver.A.2.1
	0 = The data controller did not explain or mention the cookie's definition under the PDPA scope.	


(3) Purpose of personal data collection	1 = The data controller specified the purpose of the collection, usage, and disclosure of personal data, which may or may not have a reference based on a lawful basis; however, the objectives must be clear enough to provide a framework for data processing.	Articles 19, 23(1), 24 in [27]; [50]; [51] Ver.A.2.1
	0 = The data controller did not specify the purpose of the collection, usage and disclosure of personal data or notify the unclear or ambiguous purpose.	

(4) Data processing	1 = The data controller explained how they used the collected personal data. What are the relevant processes? They must refer to a lawful basis for processing which may have more than one base to process the same data set.	Articles 19, 23(1), 24, 27 in [27]; [50]; [51] Ver.A.2.1
	0 = The data controller did not explain how the personal data was used or what relevant processes are. They might also not refer to any lawful basis for processing.	

(5) Purpose of cookie collecting	1 = The data controller clearly specified the purpose of the collection or disclosure of both necessary and non-essential cookies (if any).	Articles 19, 23(1), 24 in [27]; [50]; [51] Ver.A.2.1
	0 = The data controller did not specify the purpose of the collection, usage, and disclosure of cookies or mention an unclear purpose of the collection, usage, and disclosure of cookies.	

**2. Information disclosure / Third-party transfer**		**References**

(6) Purpose of marketing promotion	1 = In case the organization promoted direct marketing, the data controller gave direct marketing information to data subjects and did not hide other objectives.	Article 32(2) in [27]; [50]
	0 = The data controller did not mention direct marketing or notify data subjects about direct marketing.	

(7) Provide the first/ third party cookie information	1 = The data controller clarified how they collected personal data: (1) Collected directly from the data subjects (2) Received from a third party	Article 25 in [27]; [50]
	0 = The data controller did not clarify how they collected personal data: (1) Collected directly from the data subjects (2) Received from a third party	

(8) Sharing personal data with third party	1 = The data controller provided details about the third party with whom the data controller may share personal data, such as business partners, the Office of the Civil Service Commission, government agencies, and server companies.	Article 23 in [27]; [50]; [51] Ver.A.2.1
	0 = The data controller did not provide details about the third party with whom the data controller may share personal data, such as business partners, the Office of the Civil Service Commission, government agencies, and server companies.	

**3. Data retention / Storage and retention of collected data**		**References**

(9) Type of personal data collection	1 = The data controller identified all types of personal data collected from data subjects; some might have come from third parties.	Article 22 in [27]; [50]; [51] Ver.A.2.1
	0 = The data controller did not identify all types of personal data collected from data subjects; some might have come from third parties.	

(10) Storage and data retention period	1 = The data controller mentioned the retention period of personal data. It may inform how to destroy data but not specify the exact time period. However, there was an expected duration based on the purpose of usage/processing.	Article 23(3) in [27]; [50]; [51] Ver.A.2.1
	0 = The data controller did not mention or mention in ambiguous ways the retention period of personal data, including how to destroy personal data.	

(11) Where the data will be stored	1 = The data controller specified storage location, which may be in domestic or international storage.	[50]
	0 = The data controller did not specify or provide details on where the data will be stored.	

(12) Cookie retention period	1 = The data controller explained or mentioned the cookie retention period, which may be specified in the policy or the cookie description in the cookie-setting process.	Article 23(3) in [27]
	0 = The data controller did not explain or mention the cookie retention period, which may be specified in the policy or the cookie description in the cookie-setting process.	

**4. Rights of data subjects**		**References**

(13) Accessibility	1 = The data controller designed a website for easy visibility/accessibility to a privacy policy, such as providing a pop-up screen or a link to a privacy policy separated from service content.	Articles 19 23 in [27]; [50]1
	0 = The data controller did not design a website for easy visibility/accessibility to a privacy policy, such as providing a pop-up screen or a link to a privacy policy separated from service content.	


(14) Data subject's rights	1 = The data controller mentioned that data subjects are entitled to the following:	[50]; [51] Ver.A.2.1
	(1) To request access to and obtain a copy of personal data related to him or her.	
	(2) To receive personal data concerning him or her from the data controller and data subjects have the right to request the data controller to send or transfer personal data to other data controllers.	
	(3) To object to the collection, usage, and disclosure of personal data concerning him or her at any time.	
	(4) To request the data controller to erase or destroy personal data or anonymize their data to become anonymous data that cannot identify the data subjects.	
	(5) To request the data controller to restrict the use of personal data.	
	(6) To ensure that personal data remains accurate, up-to-date, complete, and not misleading.	
	(7) To be informed prior to or at the time of personal data collection regarding required details, e.g., the purpose of the collection, the data retention period, and the rights of data subjects.	
	(8) To withdraw consent at any time unless there is a restriction of the withdrawal of the consent by law or the contract which benefits data subjects.	
	(9) To file a complaint in the event that data controller/processor, including employees or service providers of the data controller/processor, violates or does not comply with PDPA or notifications issued in accordance with PDPA.	
	0 = The data controller did not mention any of the data subject's rights ((1) – (9)).	

(15) Cookie banner/ Notification of the usage of cookies	1 = The data controller provided a pop-up screen alerting when data subjects visited their websites that cookies were collected.	Article 23 in [27]; [50]
	0 = The data controller did not provide a pop-up screen alerting when data subjects visited their websites that cookies were collected.	

(16) Provide link to cookie policy	1 = The data controller provided a link to a cookie policy to facilitate data subjects to access the policy easily. The link may be placed on a cookie banner or a privacy notice page (in case a website separates a cookie policy and a privacy policy into two pages.)	Article 23 in [27]; [50]
	0 = In case there is a cookie policy separated from a privacy policy, the data controller did not provide a link to the cookie policy page to facilitate data subjects to access the policy easily.	

**5. Data Protection / Data security / Security measures**		**References**

(17) Hypertext Transfer Protocol Secure (HTTPS)	1 = There was a protocol that encrypted data or SSL (Secure Socket Layer) in the form of HTTPS (Hypertext Transfer Protocol Secure) to ensure the security of data transmission over the Internet so that data were not easily intercepted.	Article 37 in [27]
	0 = There was no protocol that encrypted data transmitted between the website and visitors. Only HTTP protocol was found on the website.	

(18) How to protect the collected personal data	1 = The data controller informed that proper security measures were in place to protect personal data, such as encryption for employees when they accessed customers' personal data.	Articles 37(1), 40 in [27]
	0 = The data controller did not inform or mention that proper security measures 40 were in place to protect personal data, such as encryption for employees when they accessed customers' personal data.	

(19) Statement notifying data subject of the limitation or scope of liability	1 = The data controllers clarified that if data subjects clicked a link to another website through this website, they should study and comply with the privacy policy appearing on that website.	[50]; [51] Ver.A.2.1
	0 = The data controller did not clarify that if data subjects clicked a link to another Ver. A.2.1 website through this website, they should study and comply with the privacy policy appearing on that website.	

**6. User Controls / User choice / Control / Opt-out options**		**References**

(20) Consent form	1 = There was a consent form which may be a checkbox or a button that was clearly separated from other contents. The format and text of the consent form were easily accessible and understandable. The contents did not deceive or mislead data subjects, including providing options to reject. The data controller did not confuse data subjects by referring to terms of service in the consent form.	Articles 19, 32 in [27]; [50]
	0 = There was no consent form with a checkbox or a button that was clearly separated from other contents. The format and text of the consent form were not easily accessible or understandable. The contents deceive or mislead data subjects, not providing options to reject. The data controller confused data subjects by referring to terms of service in the consent form.	

(21) A channel or button for users to withdraw consent easily	1 = The data controller facilitated data subjects to request withdrawal of consent. It might be an unsubscribe button or a link to a withdrawal request form that data subjects can fill out and send to the data controller, or providing information for data subjects to contact the data controller directly, or any other channels for data subjects to withdraw consent.	Articles 19, 31, 32, 33, 34 in [27]; [50]
	0 = The data controller did not facilitate data subjects to request withdrawal TDPG 3.0 of consent such as an unsubscribe button, a link to a withdrawal request form that data subjects can fill out and send to the data controller, or providing information for data subjects to contact the data controller directly, or any other channels for data subjects to withdraw consent.	


(22) Non-necessary cookie rejection	1 = The data controller provided an option to refuse the consent of collection of cookies with a button “deny, not agree, not accept, etc.” or with a cookies setting.	Articles 19, 31, 32, 33, 34 in [27]; [50]
	0 = The data controller did not provide an option to refuse the consent of collection of cookies with a button “deny, not agree, not accept, etc.”or with a cookies setting.	

(23) Cookie settings	1 = The data controller provided cookie setting options that data subjects can select whether to allow or not allow the data controller to collect non-necessary cookies, which may be classified as performance cookies, functional cookies, or cookies for targeting.	Articles 19, 31, 32, 33, 34 in [27]; [50]
	0 = The data controller did not provide a refuse option or cookie setting options that data subjects can select to allow or not allow the data controller to collect non-necessary cookies.	

(24) Non-necessary cookie consent	1 = The data controller informed that unnecessary cookies were being collected, such as performance cookies, functional cookies, and cookies for targeting.	Articles 19, 31, 32, 33, 34 in [27]; [50]
	0 = The data controller did not inform that unnecessary cookies were being collected, such as performance cookies, functional cookies, and cookies for targeting.	

(25) How to disable cookies	1 = The data controller explained how data subjects could manage cookies by setting their own browsers, which may provide information for different browsers.	[50]; [51] Ver.A.2.1
	0 = The data controller did not explain how data subjects can manage cookies by setting their own browsers.	

**7. Policy Change / Revisions in the privacy statements**		**References**

(26) If the policy changes, how data controller will do	1 = The data controller mentioned that in case of revision or update of the privacy policy or cookie policy, the data controller would notify data subjects via email, message, or other notifications.	[50]
	0 = The data controller did not mention that data subjects would be notified in case of revision or update of the privacy policy or cookie policy.	

(27) Latest revision date	1 = There was a date, month, year, or version that the privacy/cookie policy was announced appearing on the footnote or heading or somewhere in the policy.	[50]; [51] Ver.A.2.1, A.7.1
	0 = There was no date, month, year, or version that the privacy/cookie policy was announced appearing on the footnote or heading or somewhere in the policy.	

**8. Privacy contact information**		**References**

(28) Contact information	1 = The data controller gave contact information or how to contact the data controller, such as email, phone number, address, or the person responsible (Data Protection Officer: DPO) to data subjects.	Article 23(5) in [27]; [50]; [51] Ver.A.2.1, A.7.1
	0 = The data controller did not give contact information or how to contact the data controller, such as email and phone number, or the DPO to data subjects.	

**9. International and specific audiences**		**References**

(29) Protection of children's data / Child privacy	1 = The data controller mentioned the data subjects who are minors, not sui juris by marriage, or have no capacity. If consent is requested, parental consent is required.	[51] Ver.A.2.1
	0 = The data controller did not mention the data subjects who are minors, not sui juris by marriage, or have no capacity. There was no consent request.	

**10. Data categories / Collected information**		**References**

(30) Type of cookie collection	1 = The data controller described the types of cookies used on the website according to the stated purposes by categorizing cookies as both necessary and non-essential or specifying the names of cookies used.	Articles 19, 23(1) in [27]; [50]; [51] Ver.A.7.1
	0 = The data controller did not describe the types of cookies used on the website according to the stated purposes by categorizing cookies as both necessary and non-essential or specifying the names of cookies used.	

(31) Where personal data come from / How to collect	1 = The data controller specified a data collection approach, whether by obtaining it from data subjects directly or from third-party websites. The data was collected directly, such as when data subjects visited the website, purchased goods/services, and participated in any activities with the data controller. The data was collected from third-party websites such as cookies collecting personal information.	Articles 2, 25 in [27]; [50]; [51] Ver.A.2.1
	0 = The data controller did not specify or mention or provide details how to collect personal data from data subjects.	

## Data Availability

Data will be made available on request.

## Appendix A Appendix section

The scoring criteria table is provided below.

## Footnotes

1 https://www.gdpreu.org.

2 https://www.gdpreu.org/.

3 While the equal weight approach was employed in the initial version of the scoring model, there is potential for future expansion by incorporating other Multi-Criteria Decision Making (MCDM) techniques [17], [18] such as AHP [52], [53] or TOPSIS [54] to determine appropriate weights for each criterion based on input from a group of PDPA experts. However, the equal weight approach offers simplicity and ease of implementation, which is a common practice in calculating composite indices from multi-criteria in social sciences. An example of such practice is the global Multidimensional Poverty Index (MPI), which assigns equal weights to all dimensions and indicators within the same dimension [55], [56]. A *privacy principle* and a *privacy criteria* in this work are comparable to a dimension and an indicator in the global MPI, respectively.

4 In particular, their survey indicated that 62% of all MSMEs had personal data collected, but 81% of them were unaware of the PDPA.
